# Hypertensive Disorders of Pregnancy: A Systematic Review of International Clinical Practice Guidelines

**DOI:** 10.1371/journal.pone.0113715

**Published:** 2014-12-01

**Authors:** Tessa E. R. Gillon, Anouk Pels, Peter von Dadelszen, Karen MacDonell, Laura A. Magee

**Affiliations:** 1 VU medical centre, Amsterdam, the Netherlands; 2 Academic Medical Center, Amsterdam, The Netherlands; 3 Department of Obstetrics and Gynaecology, University of British Columbia, Vancouver, British Columbia, Canada; 4 Child and Family Research Institute, University of British Columbia, Vancouver, British Columbia, Canada; 5 College of Physicians and Surgeons of British Columbia, Vancouver, British Columbia, Canada; 6 Department of Medicine, University of British Columbia, Vancouver, British Columbia, Canada; 7 Department of Medicine, BC Women’s Hospital and Health Centre, Vancouver, British Columbia, Canada; University of Barcelona, Spain

## Abstract

**Background:**

Clinical practice guidelines (CPGs) are developed to assist health care providers in decision-making. We systematically reviewed existing CPGs on the HDPs (hypertensive disorders of pregnancy) to inform clinical practice.

**Methodology & Principal Findings:**

MEDLINE, EMBASE, Cochrane Central Register of Controlled Trials, Cochrane Methodology Register, Health Technology Assessments, and Database of Abstracts of Reviews of Effects (Ovid interface), Grey Matters, Google Scholar, and personal records were searched for CPGs on the HDPs (Jan/03 to Nov/13) in English, French, Dutch, or German. Of 13 CPGs identified, three were multinational and three developed for community/midwifery use. Length varied from 3–1188 pages and three guidelines did not formulate recommendations. Eight different grading systems were identified for assessing evidence quality and recommendation strength. No guideline scored ≧80% on every domain of the AGREE II, a tool for assessing guideline methodological quality; two CPGs did so for 5/6 domains. Consistency was seen for (i) definitions of hypertension, proteinuria, chronic and gestational hypertension; (ii) pre-eclampsia prevention for women at increased risk: calcium when intake is low and low-dose aspirin, but not vitamins C and E or diuretics; (iii) antihypertensive treatment of severe hypertension; (iv) MgSO4 for eclampsia and severe pre-eclampsia; (v) antenatal corticosteroids at <34 wks when delivery is probable within 7 days; (vi) delivery for women with severe pre-eclampsia pre-viability or pre-eclampsia at term; and (vii) active management of the third stage of labour with oxytocin. Notable inconsistencies were in: (i) definitions of pre-eclampsia and severe pre-eclampsia; (ii) target BP for non-severe hypertension; (iii) timing of delivery for women with pre-eclampsia and severe pre-eclampsia; (iv) MgSO4 for non-severe pre-eclampsia, and (v) postpartum maternal monitoring.

**Conclusions:**

Existing international HDP CPGs have areas of consistency with which clinicians and researchers can work to develop auditable standards, and areas of inconsistency that should be addressed by future research.

## Introduction

The hypertensive disorders of pregnancy (HDPs) are common, complicating up to 6–8% of all pregnancies. As such, the HDPs are a leading cause of maternal and perinatal mortality and morbidity, worldwide. It is anticipated that this situation will only worsen, given the rising prevalence of obesity and metabolic syndrome among women of childbearing age [1]–[2].

Many national and international clinical practice guidelines (CPGs) have been published on the diagnosis, evaluation, and management of the HDPs. Although many such CPGs have quoted the same research papers, the between-guideline variability in specific recommendations has been highlighted by international multicentre research endeavours, such as the CHIPS Trial (Control of Hypertension In Pregnancy Study) [3]. However, no analysis of CPG quality and consistency has been achieved as for other conditions [4]–[9]. In addition, substandard care of women with pregnancy hypertension, especially failures related to diagnosis, evaluation, and management, continues to be recognised as a contributor to maternal death in well- [10]–[11] and less-resourced settings [12].

We sought to review published CPGs covering the diagnosis, evaluation, and management of the HDPs, in order to inform practicing clinicians about the consistency of the recommendations and the quality of the source guidelines.

## Methods

### Eligibility

Included were multi-disciplinary CPGs that were: (i) published within the last 10 years (2003–13), and the then accepted 2014 SOGC guideline (now published) [30], [31], (ii) covered the diagnosis, assessment and management of one/more of the HDPs in human pregnancy, and (iii) were written in English, French, Dutch or German (i.e., languages understood by the review authors). Excluded were CPGs that: were adapted for local use from an existing CPG, had no references, or were not regional/national/international in scope.

### Literature search

A comprehensive literature review was undertaken by a librarian (KM) of the College of Physicians and Surgeons of British Columbia, in consultation with the principal authors of this article. Key words, related to hypertension, pregnancy, and guidelines, were used to search MEDLINE, EMBASE, Cochrane Central Register of Controlled Trials, Cochrane Methodology Register, Health Technology Assessments, and Database of Abstracts of Reviews of Effects using the Ovid interface (Appendix S1). As not all CPGs may be available on bibliographic databases cited above, additional sources were searched including personal records. Grey Matters, a tool for evidence-based searching on the internet developed by the Canadian Agency for Drugs and Technologies in Health, was used to locate online grey literature sources, which were searched using key words such as “hypertensive disorders of pregnancy”, “gestational hypertension”, “hypertension during pregnancy”, “pregnancy induced hypertension”, and “hypertension gestationnelle”; this site includes the National Guidelines Clearinghouse. Similar search terms combined with “guideline” or “recommendation” were entered on Google Scholar and the first 100 results were screened, considering most relevant results appear first. Finally, the national websites of societies of obstetrics and gynaecology of the main French-, English-, Dutch- or German-speaking countries were searched.

### Processing data

The AGREE II tool was used to assess the methodological quality of all included CPGs [13]. Using the standardised AGREE II methodology, scores of 1 to 7 were given both overall and to each of 23 items in six domains related to standard methodology. Percentages of maximum possible scores were calculated for each domain. Also, each reviewer responded to the following question, “I would recommend this guideline for use” with ‘yes’, ‘yes with modifications’ and ‘no’. For this review, ‘yes’ was given to a guideline considered useful as a reference document for busy clinicians as such, ‘no’ was assigned to CPGs that did not formulate recommendations (but consisted of text only). Two authors (TG, AP, and/or LM) rated each CPG independently and discrepancies were resolved by consensus.

Descriptive analyses were undertaken to present the general characteristics of each CPG, including the grading system used to assess the quality of evidence and strength of recommendations. For all CPGs recommended for use, we examined: (i) criteria for diagnosis and HDP classification, using information from the tables and text as diagnostic criteria do not lend themselves well to recommendations, and (ii) recommendations about ‘actionable items’ related to prevention of pre-eclampsia or management of any HDP, that were reported commonly (by at least three CPGs) and/or designated to have a high rating for quality of evidence and strength of recommendation.

This was an analysis of published data and did not require research ethics board approval.

## Results

### Literature search and guideline selection

**Figure 1:**
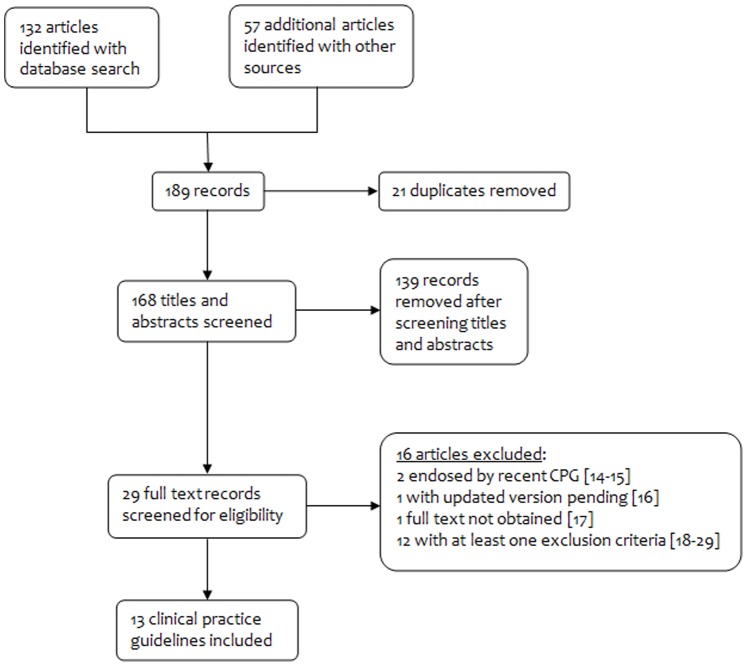
Search results.

Figure 1 shows that our search strategy yielded 189 records for consideration, 132 from database searches and 57 identified through other sources. Following screening and review of full text papers, 16 articles were excluded [14]–[29] and there were 13 CPGs for inclusion in addition to the 2014 ISSHP position statement.

### Guideline characteristics

Table 1 presents general characteristics of the included CPGs, developed in Canada (Society of Obstetricians and Gynaecologists of Canada (SOGC), Association of Ontario Midwives (AOM)) [30]–[32], the United Kingdom (National Institute for Health and Clinical Excellence (NICE), pre-eclampsia community guideline (PRECOG), PRECOG II) [33]–[35], the United States of America (American College of Obstetricians and Gynecologists (ACOG), American Society of Hypertension (ASH)) [36]–[37], Australia (Queensland Maternity and Neonatal Clinical Guidelines Program (QLD)) [38]–[39], the Netherlands (Nederlandse Vereniging voor Obstetrie en Gynaecologie (NVOG)) [40], and Germany (Deutschen Gesellschaft fur Gynakologie und Geburtshilfe (DGGG)) [41]. Most CPGs were national (8/13), but three were multinational, from Australasia (Society of Obstetric Medicine of Australia and New Zealand (SOMANZ)) [42], the World Health Organization (WHO) [43], and the European guideline for cardiovascular diseases (ESC) [44]. Most CPGs (8/13) were new, but five were updates of previous CPGs published 6–13 yr prior. All but two guidelines [NICE, WHO] had professional organizations behind them. The number of pages (including appendices) varied from 3 [PRECOG II] to 1188 [NICE] and the number of recommendations from 7–150 in the 10 CPGs that made formal recommendations. Three CPGs [PRECOG, PRECOG II, AOM] were written specifically for community [PRECOG, AOM] or hospital-based [PRECOG II] midwifery care.

**Table 1:** General description of included Clinical Practice Guidelines.

Domain andsub-questions	PRECOG*	DGGG	SOMANZ	ASH	PRECOGII*	QLD	NICE	WHO	ESC	NVOG	AOM*	ACOG	SOGC
	2005	2007	2008	2008	2009	2010	2010	2011	2011	2011	2012	2013	2014
**Status of the CPG**													
New	**√**	**–**	**√**	**√**	**√**	**√**	**√**	**√**	**√**	**–**	**–**	**–**	**–**
Update of previous	–	**√**	–	–	–	–	–	–	–	**√**	**√**	**√**	**√**
* N yr since prior CPG*	–	6								6	11	13	6
**Level of development**													
International			**√**					**√**	**√**			–	
National	**√**	**√**		**√**	**√**		**√**			**√**		**√**	**√**
Regional						**√**					**√**	–	
**Organization** **behind CPG**													
Professional	**√**	**√**	**√**	**√**	**√**	**√**			**√**	**√**	**√**	**√**	**√**
Government							**√**						
Other								**√**					
**Usability**													
N pages	5	16	31	12	3	32	1188	40	51	103	46	100	63
* Body of document*	5	16	31	12	3	19	216	33	4	66	46	100	40
* Appendices*	–	0	0	0	0	13^Δ^	972	7	0	37	–	–	23^†^
N references	24	260	161	69	17	20	277	34	254	105^§^	136	328^§^	535
N recommendations	?¤	0	0	0	27	11	123	23	7	17	19	60	150
**Funding stated**	**√**	–	–	–	**√**	**√**	**√**	**√**	**√**	**√**	**√**	–	**√**

*Guidelines developed for community/midwifery use, ^Δ^ The supplement was considered as an appendix, ^†^ The executive summary was regarded as an appendix, ^§^ Represents all references for all chapters and includes duplicates, ¤ Recommendations presented in 3 boxes and 2 tables.

All CPGs covered pre-existing (chronic) hypertension, gestational hypertension, and preeclampsia, with the exception of the WHO guideline that focused only on pre-eclampsia and eclampsia. Six CPGs mentioned white coat hypertension [SOMANZ, QLD, NICE, AOM, ACOG, SOGC]. Only SOGC mentioned reversed white coat effect [SOGC].

### Assessment of the evidence and strength of recommendations

Two CPGs did not grade the quality of evidence [SOMANZ, ASH]. Table 2 shows that the other 10 CPGs used eight different systems to grade the quality of the evidence: GRADE (N = 3) [WHO, SOGC, ACOG], the Canadian Task Force on Preventive Health Care (N = 3) [SOGC, AOM, QLD], or a novel system (N = 4) [ESC, DGGG, PRECOG and PRECOG II, NICE and NVOG], two of which classified diagnostic accuracy and intervention studies using different criteria [NICE, NVOG]. SOGC used both GRADE and the Canadian Task Force on Preventive Health Care. Meta-analysis of randomised controlled trials (RCTs) was rated among the highest quality evidence by all but the Canadian Task Force on Preventive Health Care which does not mention this study design. The rating used by NICE had three levels of high quality evidence, whereas most other systems had one. All systems included expert opinion or consensus among the lowest quality of evidence, although two systems included descriptive studies as well (PRECOG, and PRECOG II; Canadian Task Force on Preventive Health Care).

**Table 2:** Grading systems for assessing the levels of evidence*.

	PRECOGPRECOG II DGGG	Canadian TaskForce ForPreventive HealthCare	NICE (intervention studies)	NICE(accuracy ofdiagnostic tests)	ESC	NVOG(methodological quality of studies)	NVOG(level of evidenceof conclusions)	GRADE
**Highest** **possible** **level**	**Ia (meta-analysis of RCTs)**	**I** † **(1 or more RCT)**	**1++ (very low risk bias meta-analysis or** **systematic review of RCTs or RCTs)**	**Ia (systematic review of** **level-1 studies)**	**A (meta-analysis** **of RCTs or RCTs)**	**A1 (systematic reviews or** **meta-analysis of RCTs)**	**1 (1 systematic review or** **meta-analysis of RCTs, or 2 RCTs)**	**High**
	**Ib (1 or more RCT)**		**1+ (low risk bias meta-analysis or** **systematic review of RCTs or RCTs)**			**A2 (RCTs)**		
			**1− (high risk bias meta-analysis or** **systematic review of RCTs or RCTs)**					
	IIa	II-1	2++	Ib	B	B	2	Moderate
	IIb	II-2	2+	II			3	Low
		II-3	2−	III				
*Lowest* *possible* *level*	*III (non-experimental* *descriptive studies)*		*3 (non-analytical studies)*			*C (non-comparative studies)*		
	*IV (Expert* *report/opinion)*	*III (Expert report/opinion)*	*4 (Expert consensus/opinion)*	*IV (Expert consensus/opinion)*	*C* ‡ *(Expert opinion, small* *studies, retrospective studies)*	*D (Expert opinion)*	*4 (Expert opinion)*	*Very low*

*Bold indicates comparable gradings for ‘high quality’ evidence. Italic indicates comparable shading for ‘low quality’ evidence.

† Does NOT include meta-analysis of RCTs.

‡ includes small studies and retrospective studies as well.

Table 3 shows that the strength of the recommendations was presented by seven CPGs using four approaches: GRADE (N = 3) [WHO, SOGC, ACOG], the Canadian Task Force on Preventive Health Care (N = 3) [SOGC, AOM, QLD], or a novel system (N = 2) [ESC, PRECOG and PRECOG II]; SOGC used both GRADE and the Canadian Task Force on Preventive Health Care.

**Table 3:** Grading systems for assessing the strength of recommendations.

PRECOG PRECOG II(grade given according tolevel of evidencerecommendation wasbased on)	Canadian Task ForceFor PreventiveHealth Care	NICE(intervention studies)	NICE(accuracy ofdiagnostic tests	ESC	NVOG(methodologicalquality of studies)	NVOG (level ofevidenceof conclusions)	GRADE
Grade A	A (good evidence to recommend)	–	–	Class I (treatment/procedurebeneficial, useful, effective)	–	–	Strong
Grade B	B (fair evidence torecommend)	–	–	Class II (evidence conflictingabout usefulness/efficacy)	–	–	Weak
Grade C	C (conflicting evidence,does not allow to makerecommendation)	–	–	Class IIa (evidence in favourof usefulness/efficacy)	–	–	
Grade D	D (fair evidence toNOT recommend)	–	–	Class IIb (usefulness/efficacyless well established)	–	–	
GPP	E (good evidence toNOT recommend)	–	–	Class III (treatment/procedureNOT useful/effective)	–	–	
	I (insufficient evidence,does not allow to makerecommendation)	–	–		–	–	

Two guidelines rated neither the quality of evidence nor the strength of their recommendations [SOMANZ, ASH]. Two CPGs rated the quality of evidence without rating the strength of their recommendations [NICE, NVOG], and one rated evidence discussed in the text without making recommendations [DGGG].

### Scope of the guidelines

All CPGs covered pre-existing (chronic) hypertension, gestational hypertension, and preeclampsia, with the exception of the WHO guideline that focused only on pre-eclampsia and eclampsia. Five CPGs mentioned white coat hypertension [SOMANZ, QLD, AOM, ACOG, SOGC]. Only SOGC mentioned reversed white coat effect [SOGC].

### AGREE II scoring

The AGREE II scores for each CPG are presented in Table 4. The highest scores (≧80%) were obtained for the domains ‘scope and purpose’ (N = 5 CPGs) [PRECOG, QLD, NICE, WHO, SOGC] and ‘clarity of presentation’ (N = 6) [NICE, WHO, NVOG, AOM, ACOG, SOGC] for which three CPGs with text only had low scores [ASH, DGGG, SOMANZ]. The lowest scores were obtained in the domains of: (i) ‘applicability’ (as only one CPG met most criteria for presenting facilitators and barriers for CPG implementation [WHO] and only three listed auditing or monitoring criteria [SOMANZ, NICE, WHO]), (ii) ‘editorial independence’ (as most CPGs were funded/initiated by professional organisations and only three CPGs stated that the funding body had not influenced CPG content [PRECOG II, NVOG, AOM]), and (iii) ‘stakeholder involvement’ because the views and preferences of the target population were usually not represented. No CPG achieved ≧80% of the maximal score for all six domains, but the WHO and NICE guidelines did so for 5/6 domains. Four guidelines did not obtain one score ≧80% in any domain [ASH, DGGG, ESC, SOMANZ]; these same CPGs were also rated as not being clinically useful. As such, the HDP classification and recommendations regarding prevention and treatment are described for the remaining nine guidelines.

**Table 4:** Clinical Practice Guideline domain scores using the AGREE-II instrument, with details of content included where relevant.

Domain and sub-questions	PRECOG2005	DGGG2007	SOMANZ2008	ASH2008	PRECOG II 2009	QLD2010	NICE2010	WHO2011	ESC2011	NVOG2011	AOM2012	ACOG2013	SOGC2014
**1. Scope and purpose**	83%	6%	33%	67%	78%	89%	100%	100%	44%	78%	72%	44%	100%
* Pre-existing (chronic) hypertension*	**√**	**√**	**√**	**√**	**√**	**√**	**√**	–	**√**	**√**	**√**	**√**	**√**
* Gestational hypertension*	**√**	**√**	**√**	**√**	**√**	**√**	**√**	–	**√**	**√**	**√**	**√**	**√**
* Pre-eclampsia*	**√**	**√**	**√**	**√**	**√**	**√**	**√**	**√**	**√**	**√**	**√**	**√**	**√**
* White coat hypertension*	–	–	**√**	–	–	**√**	–	–	–		**√**	**√**	**√**
* Reversed white coat hypertension*	–	–	–	–	–	–	–	–	–	–	–	–	**√**
**2. Stakeholder involvement**	61%	17%	11%	17%	61%	61%	89%	83%	33%	94%	44%	28%	78%
**3. Rigour of development**	27%	29%	19%	10%	17%	44%	90%	94%	63%	73%	46%	35%	73%
Assessment of evidence	**√**	**√**	–	–	**√**	**√**	**√**	**√**	**√**	**√**	**√**	**√**	**√**
**4. Clarity of presentation**	78%	22%	44%	33%	61%	61%	89%	94%	67%	89%	89%	94%	89%
**5. Applicability**	33%	0%	25%	0%	71%	33%	88%	79%	29%	21%	17%	17%	46%
**6. Editorial independence**	25%	0%	0%	0%	75%	25%	50%	50%	42%	100%	50%	0%	25%
**Overall score** *	4/7	3/7	4/7	3/7	3/7	4/7	6/7	7/7	4/7	6/7	5/7	5/7	6/7
**CPG recommended for use**	yes	no	no	no	Yes withmodifications	yes	yes	yes	no	yes	yes	yes	yes

*Scores represent the consensus of both reviewers, following independent assessment and discussion (numbers indicate scores and proportions are of the total score for each domain).

### Definitions/classification (Tables S1 and S2)

Hypertension is defined according to systolic and diastolic BP (≧140/90 mmHg) (N = 5) [QLD, NICE, NVOG, ACOG, SOGC], diastolic BP alone (≧90 mmHg) (N = 3) [PRECOG, PRECOG II, AoM], or not at all (N = 1) [WHO] (Table S1). Severe hypertension is defined in 7/9 CPGs, as BP≧160/110 mmHg (N = 6) [NICE, QLD, NVOG, AOM, ACOG, SOGC] or ≧170/110 mmHg [PRECOG II].

Screening for proteinuria is specifically advocated only by four CPGs for women with a HDP [AOM, NICE, PRECOG, SOGC]; when performed, testing methods should be by dipstick (visual [PRECOG, AOM], automated [NICE], or either [SOGC]), but NICE advocates using a random urine protein:creatinine ratio (PrCr) in a secondary care setting [NICE] (Table S1). Significant thresholds for proteinuria are: ≧1+ [PRECOG, SOGC] or ≧2+ [PRECOG II, QLD], with two CPGs specifying that a threshold of ≧1+ should be used only when there is associated hypertension [PRECOG II] or other manifestations of pre-eclampsia [AOM].

For quantification of proteinuria, criteria are: “dipstick” ≧1+ [AOM], random urine PrCr ≧30 mg/mmol [PRECOG, PRECOG II, NICE, SOGC], and/or 24 hr urinary protein ≧0.3 g/d [PRECOG, PRECOG II, NICE, NVOG, ACOG SOGC] (with completeness of the urine collection emphasised by two CPGs [NICE, SOGC]).

There is consistency with regards to the definitions of chronic (pre-existing) and gestational hypertension (Table S1). Chronic hypertension predates pregnancy or is documented before 20 wks. One CPG specifies that this must be essential (i.e., without known cause) [QLD]) and three list either secondary causes and/or co-morbid conditions that would influence decisions about BP control [AOM, QLD, SOGC]. Gestational hypertension is new hypertension that develops at or after 20 wks; although implied by all CPGs, some specify that there must be neither proteinuria [QLD] nor other features of pre-eclampsia (N = 2) [ACOG, NICE] Three CPGs specify that BP must return to normal postpartum, at 12 wks (N = 2) [QLD, NVOG] or at an unspecified time [ACOG].

All CPGs define pre-eclampsia as gestational hypertension with proteinuria which is more often a mandatory criterion (N = 5) [PRECOG, PRECOG II, WHO, NICE, NVOG] than not (n = 4) [AOM, QLD ACOG, SOGC] (Tables S1 and S2). Two CPGs specify that the proteinuria must resolve after delivery [PRECOG, PRECOG II]. Although four also include gestational hypertension with one/more systemic feature of pre-eclampsia, there is no consistency with regards to those features that include fetoplacental abnormalities and/or maternal symptoms, signs, and abnormal laboratory findings [ACOG, AOM, QLD, SOGC]. The most common maternal manifestations listed are: headache/visual symptoms (N = 4 CPGs), right upper quadrant/epigastric abdominal pain (N = 3), severe hypertension (N = 2), eclampsia (N = 2), pulmonary oedema (N = 3), low platelets (N = 4), elevated serum creatinine (N = 4), and elevated liver enzymes (N = 4); only one CPG specifies hyperreflexia. Fetal manifestations of pre-eclampsia are specified by three CPGs, all of which list IUGR (not defined) (N = 3) and abruption without evidence of foetal compromise (N = 3); one specifies stillbirth.

‘Superimposed’ pre-eclampsia is not clearly defined. Three CPGs do not address this at all, and six define it variably as worsening hypertension (N = 3) [AOM, ACOG, SOGC], new/worsening proteinuria (N = 3) [AOM, ACOG, SOGC] or one/more other systemic features (N = 4) [NVOG, AOM, ACOG, SOGC]. ‘Worsening’ hypertension is defined clearly by two CPGs as either: (i) a sudden increase in BP or the need to increase antihypertensive dose [ACOG], or (ii) the need for three antihypertensive medications for BP control at ≧20 weeks [SOGC]. Proteinuria is a mandatory criterion according to ACOG (Table S1).

‘Severe’ pre-eclampsia is defined by most (7/9) CPGs, but there is little consistency. Heavy proteinuria is included by some (N = 3) [WHO, NVOG, AOM], but specifically excluded by others (N = 2) [ACOG, SOGC]. Five CPGs define end-organ complications of severe pre-eclampsia; the most common maternal are: headache/visual symptoms (N = 5 CPGs), right upper quadrant/epigastric abdominal pain (N = 4), severe hypertension (N = 5), eclampsia (N = 2), pulmonary oedema (N = 3), low platelets (N = 4), renal insufficiency (N = 3), and elevated liver enzymes (N = 3); these mirror the diagnostic criteria used in some guidelines. Fetal manifestations of pre-eclampsia are specified by three CPGs, all of which list stillbirth and none of which specify abruption without evidence of fetal compromise; IUGR is included by WHO and SOGC, but specifically excluded by ACOG. The SOGC ‘severity’ criteria are indications for delivery, and include some features that in other CPGs: (i) define pre-eclampsia but not severe pre-eclampsia (e.g., stroke), (ii) define both pre-eclampsia and severe pre-eclampsia (e.g., eclampsia, pulmonary oedema, platelet count <100×10^9^/L, and acute kidney injury), or (iii) define neither pre-eclampsia nor severe pre-eclampsia but are widely regarded as indications for delivery (e.g., uncontrolled severe hypertension).

In the three CPGs that specify that proteinuria is mandatory to define pre-eclampsia [WHO, NICE, NVOG], severe pre-eclampsia is the development of: (i) pre-eclampsia at <34 wk [WHO], or (ii) one/more features of end -organ dysfunction that is either not defined [WHO and NICE] or listed as “symptoms” [NVOG], heavy proteinuria [NVOG, WHO], or severe hypertension [NVOG, WHO] (Table S2).

In the four CPGs that do not include proteinuria as mandatory to define pre-eclampsia [AOM, QLD ACOG, SOGC], severe pre-eclampsia is the development of: (i) pre-eclampsia at <34 wk [AOM], (ii) proteinuria *plus* one/more features that alone would signify pre-eclampsia (cerebral/visual disturbances, pulmonary oedema, platelet count <100×109/L, renal insufficiency, or elevated liver enzymes) [ACOG], or (iii) one/more features of end-organ dysfunction described as: heavy proteinuria [AOM], one/more features of HELLP [QLD], new persistent and otherwise unexplained right upper quadrant/epigastric abdominal pain [ACOG], severe hypertension [AOM ACOG], or those dysfunctions requiring delivery [SOGC] (Table S2).

Eclampsia is consistently defined by new onset and otherwise unexplained seizures in the setting of pre-eclampsia (N = 5 CPGs) [NICE, QLD, WHO, ACOG, SOGC]. No guideline defines the widely used term, ‘imminent eclampsia’.

### Prediction (Table S3)

Screening only by clinical risk markers is recommended (N = 3 CPGs, 0 high rating), with no guideline recommending routine use of biomarkers or ultrasonography. The actual risk markers used were not reviewed.

Table 5 presents information from the two guidelines that present recurrence risks for gestational hypertension and pre-eclampsia according to their occurrence in the prior pregnancy [NICE, SOGC].

**Table 5:** Risks of recurrence for GH and PET reported in the NICE and SOGC guidelines.

	Second pregnancy* †	
First/prior pregnancy	GH	PET
**GH**	16–47% (NICE)	2–7% (NICE)
	Median 21% (SOGC)	Median 4% (SOGC)
**PET**	13–53% (NICE)	16% for PET (NICE)¤ §
	Median 22% (SOGC)	Median 15% (SOGC)

*Recurrence more likely in women with higher BMI, and when the prior PET was: of early onset, “severe”, or complicated by eclampsia or HELLP syndrome (SOGC).

† The following traditional PET risk markers for first occurrence do NOT influence recurrence: multiple gestation, change of partner, and long interpregnancy interval (SOGC).

¤ 25% if complication of PET let to birth <34 weeks (NICE).

§ 55% if complications let to birth <28 weeks (NICE).

### Prevention (Table S4)

Women at low risk of pre-eclampsia are recommended NOT to restrict dietary salt [N = 4, 0 high rating] [ACOG, NICE, SOGC, WHO], or take vitamins C and/or E (N = 4, 3 high rating) [ACOG, NICE, SOGC, WHO] or diuretics (N = 3, 1 high rating) [NICE, SOGC, WHO]. Of interest, few guidelines commented on calcium supplementation (1–2 g/d) if women have low calcium intake (N = 2, not recommended, 1 high rating) [WHO, SOGC] or low-dose aspirin (1, not recommended, 1 high rating) [SOGC].

Women at increased risk of pre-eclampsia are recommended to take calcium supplementation (1–2.5 g/d) if they have low calcium intake (N = 3 CPGs, 2 high rating) [AOM, WHO, SOGC], and low-dose aspirin (60–162 mg/d) (N = 5 CPGs, 2 high rating) [ACOG, AOM, NICE, SOGC, WHO]. Aspirin is recommended to be taken from early pregnancy (N = 5, 1 high rating) [ACOG, AOM, NICE, SOGC, WHO] until delivery (N = 3, 1 high rating) [AOM, NICE, SOGC]. These women are recommended NOT to restrict dietary salt (N = 3, 0 high rating) [ACOG, NICE, WHO] or take vitamins C and/or E (N = 4, 3 high rating) [ACOG, NICE, SOGC, WHO].

### Treatment (Table S5)

#### Bed rest

No consistent (or high rating) recommendations are made about bed rest by type of HDP (N = 4 CPGs) [NICE, WHO, ACOG, SOGC]. Bed rest is NOT recommended for any HDP with two exceptions: gestational hypertension for which bed rest in hospital (vs. unrestricted activity at home) may be useful [SOGC], and severe pre-eclampsia which is excluded from the ACOG rest recommendations.

#### Admission to hospital

The only indication for hospital admission that is consistently recommended is severe hypertension (N = 5 CPGs, 0 high rating) [QLD, NICE, PRECOG, SOGC].

#### Antihypertensive therapy

Six CPGs discuss antihypertensive therapy [ACOG, QLD, NICE, NVOG, SOGC, WHO].

Severe hypertension should be treated (N = 6, 1 high rating), but BP goals to achieve vary: <150/80–100 mmHg [NICE] or <160/110 mmHg (N = 4, 1 high rating) [QLD, ACOG, SOGC] for all but women with chronic hypertension, for whom ACOG recommends achieving a BP<160/105 mmHg [ACOG]. Recommended drugs of first choice are intravenous (iv) labetalol (N = 3, 1 high rating for iv) [NICE, NVOG, SOGC], oral nifedipine (N = 3, 1 high rating) [NICE, NVOG, SOGC], and iv hydralazine (N = 2, 1 high rating) [NICE, SOGC]; two CPGs leave the choice to the clinician [QLD, WHO]. MgSO_4_ should not be used as an antihypertensive (N = 1, high rating) [SOGC].

Target BP for women with non -severe hypertension is variable (N = 4 CPGs, 0 high ratings), and dependent on associated co-morbidities and/or type of HDP. For women with end-organ dysfunction that can be exacerbated by elevated BP, treatment to BP<140/90 mmHg is recommended [NICE, SOGC]. For women without target-organ damage, treatment targets are: (i) for any HDP, <150/80–100 mmHg [NICE], 130–159/80–105 mmHg [SOGC], or <160/110 mmHg [NVOG], (ii) for women with chronic hypertension, 120–159/80–104 mmHg [ACOG], and (iii) for women with gestational hypertension or non-severe pre-eclampsia, <160/110 mmHg [ACOG]. Oral methyldopa (N = 4, 1 high rating) [NICE, NVOG, ACOG, SOGC], oral labetalol (N = 4, 1 high rating) [NICE, NVOG, ACOG, SOGC], and nifedipine (N = 4, 1 high rating) [NICE, NVOG, ACOG, SOGC] are most commonly recommended, although SOGC also lists ‘other calcium channel blockers’ as an option with a high rating. Antihypertensives NOT to use are ACE inhibitors and ARBs (each N = 4, 0 high rating).

For women with chronic hypertension who are taking antihypertensive therapy and planning pregnancy, it is recommended that preconceptual counselling be undertaken (N = 4) [NICE, QLD, NVOG, SOGC] and that this include discussion of alternatives to ACE inhibitors and ARBs which should be stopped if inadvertently taken in early pregnancy (N = 4) [NICE, NVOG, ACOG, SOGC].

#### Antenatal corticosteroids

Specific recommendations for women with HDPs are made by four CPGs, by gestational age, time to delivery, and/or type of HDP. Although all recommend steroids at “<34 wk”, there is some imprecision in how that is defined: “to 34 wk” [NICE], “before 34 wk” [NVOG], ≤33+6 and ≤34+0 wk in the same CPG [ACOG], and ≤34+6 wk [SOGC]. Three CPGs recommend antenatal corticosteroids for HDPs that may require delivery within the next 7 days [NVOG, NICE, SOGC]. Antenatal corticosteroids are recommended specifically for all women with pre-eclampsia (N = 1 high rating) [SOGC], superimposed PET (N = 1 high rating) [ACOG], or severe pre-eclampsia who are undergoing expectant care (N = 1 high rating) or require delivery within the next 48 hr (N = 1, 0 high rating) [ACOG].

Corticosteroids are NOT recommended to improve clinical outcomes in HELLP syndrome (N = 4, 0 high rating) [ACOG, NICE, SOGC, WHO], but one of these CPGs [ACOG] suggested considering this therapy if an improvement in platelet count would be useful.

#### Timing of delivery

Recommendations for delivery (and administration of antenatal corticosteroids, if appropriate) focus on women with pre-eclampsia (N = 5 CPGs) [ACOG, NICE, NVOG, SOGC, WHO]. Uncontrolled severe hypertension is the most widely regarded maternal indication for delivery (and treatment) (N = 3, 0 high rating) [NICE, WHO, ACOG]. Expectant care is considered appropriate depending on the type of HDP and gestational age, assuming that women and fetuses can be appropriately managed and cared for when delivered.

Women with pre-eclampsia can be expectantly managed at <34 wk (N = 3, 0 high rating) [NICE, ACOG, SOGC], but they should be delivered at term (N = 4, 1 high rating) [NICE, WHO, ACOG, SOGC]). If pre-eclampsia is severe, women should be delivered if they are prior to fetal viability (N = 3, 1 high rating for HELLP) [WHO, ACOG, SOGC] or if they are at term (N = 4, 1 high rating) [NICE, WHO, ACOG, SOGC]. Women with gestational hypertension should be delivered at term (N = 3, 0 high rating) [WHO, ACOG, SOGC]. There is no consistent guidance for women with chronic hypertension.

#### Labour and delivery

Issues related to labour and delivery were addressed by 5/9 CPGs [ACOG, AOM, QLD, NICE, SOGC]. Without fetal compromise, mode of delivery should be based on the clinical circumstances and usual obstetric indications (N = 4, 0 high rating) [ACOG, QLD, NICE, SOGC]. If a vaginal delivery is planned, and the cervix is unfavourable, cervical ripening should be undertaken (N = 2, 2 high rating) [QLD, SOGC]. Active management of the third stage of labour is recommended with oxytocin (N = 2, 2 high rating) [AOM, SOGC].

#### Magnesium sulphate (MgSO4) indications

MgSO_4_ is indicated for treatment of eclampsia (N = 6, 3 high rating) [NICE, QLD, NVOG, WHO, ACOG, SOGC] and severe pre-eclampsia (N = 5, 3 high rating) [NICE, NVOG, WHO, ACOG SOGC] although the ACOG CPG specified only intrapartum and postpartum administration. There was less certainty about recommending MgSO_4_ for non-severe pre-eclampsia (N = 3, 0 high rating) [NVOG, ACOG, SOGC] although no CPG recommended against it.

#### Postpartum

Many guidelines made recommendations that immediately postpartum, BP may increase (N = 3, o high rating) [NICE, ACOG, SOGC] and pre-eclampsia may worsen or appear for the first time (N = 5, 0 high rating) [AOM, NICE, QLD, ACOG, SOGC]. Antenatal antihypertensive therapy should be continued (N = 3, 0 high rating) [NICE, SOGC, WHO]; no guideline recommended that it be stopped completely. Although the treatment of severe hypertension followed similar recommendations to those for women before delivery (see ‘Antihypertensive therapy’) (N = 4, 1 high rating) [NICE, WHO, ACOG, SOGC], treatment targets for non-severe hypertension were generally lower: for women with chronic hypertension, <140/90 mmHg [NICE, SOGC] or <150/100 mmHg [ACOG], for women with GH, <150/100 mmHg [NICE, ACOG], and for women with pre-eclampsia, <150/100 mmHg [NICE, ACOG] (none of high rating). CPGs reflected the association between the HDPs and future health (with regards to hypertension, renal disease, and other long-term cardiovascular disease), and suggested lifestyle counselling) (N = 5, 1 high rating for achieving a health BMI among obese women) [ACOG, AOM, QLD, NICE, SOGC].

### Other

Some CPGs present detailed information about an area not covered by others and, therefore, were not discussed above. Examples include detailed information about anaesthesia/analgesia [SOGC], maternal monitoring and transfer of care from midwifery to secondary care settings [PRECOG, PRECOG II, NICE], or postpartum transfer back to the community [NICE] (Table S5).

## Discussion

### Findings

We identified 13 CPGs that published recommendations about the diagnosis, classification, prevention and treatment of the HDPs. Four CPGs were assessed as ‘not useful’ to busy clinicians, based on text-only publication [DGGG, SOMANZ, ASH] or limited text with only a few focused recommendations [ESC]. Analysis of the nine remaining CPGs revealed few consistencies and/or high rating recommendations. Consistency was seen for the definitions of hypertension, proteinuria, chronic and gestational hypertension. Consistency and high ratings (by at least one CPG) was seen for: (i) the preventative strategies of calcium (in the setting of low intake) and low-dose aspirin for women at increased risk of pre-eclampsia, and neither vitamins C and E or diuretics; (ii) antihypertensive treatment of severe hypertension; (iii) MgSO_4_ for eclampsia and severe pre-eclampsia; (iv) antenatal corticosteroids at <34 wks when delivery is probable within the next seven days; (v) delivery for women with severe pre-eclampsia who do not yet have a viable fetus and for those with any pre-eclampsia at term; and (vi) active management of the third stage of labour with oxytocin.

Notable inconsistencies, illustrative of a lack of consensus, were in areas well reported by CPGs that differed nevertheless in their recommendations: (i) definitions of pre-eclampsia and in particular, severe pre-eclampsia and superimposed pre-eclampsia that reflect our evolving understanding of the multisystem nature of the disease; (ii) target BP among women with non-severe hypertension, regardless of the HDP; (iii) timing of delivery for women with pre-eclampsia and severe pre-eclampsia; (iv) MgSO4 for non-severe pre-eclampsia, and (v) postpartum monitoring for maternal safety and improvement of long-term cardiovascular health. These are areas requiring further research and consensus-building for optimising management of a high risk group of women.

Some guidelines covered areas neglected by others, and those CPGs could be useful sources of specific information. Notable examples include post-delivery discharge planning for transfer of care [NICE] and obstetric anaesthesia for the HDPs [SOGC].

### How our findings fit with the published literature

We are aware of only one review of CPGs for pregnancy hypertension [45]. The AGREE II instrument was used to evaluate methodological quality; CPGs scored highest in “*clarity of presentation*” and lowest in “*editorial independence”,* consistent with our findings. Between-CPG differences in the number and extensiveness of recommendations were identified, but recommendation content, similarities and differences between guidelines were not explored.

The 2014 position statement from the International Society for the Study of Hypertension in Pregnancy (ISSHP) endorses areas of consistency within published CPGs [46]. Of note, ISSHP endorses a definition of pre-eclampsia that does not require proteinuria, but can be made based on maternal end-organ involvement and/or fetal IUGR. Uniquely, ISSHP does not support a distinction between severe and “mild” pre-eclampsia which, “…should be considered as one that is at anytime capable of being severe and life-threatening for mother and baby”. Superimposed pre-eclampsia should not be diagnosed based on a rise in BP alone. Gestational proteinuria is mentioned specifically as potentially signifying evolving pre-eclampsia or underlying renal disease. All women with pre-eclampsia should be admitted to hospital, at least initially. MgSO_4_ is advocated for all women with pre-eclampsia in low-and-middle-income countries. Particular emphasis is placed on the importance of recognising ‘white coat’ hypertension, the promising future of biomarkers as diagnostic and/or prognostic tools [47], and the importance of each unit having its own written policies to promote uniform care, the outcomes of which can be monitored.

Two of the CPGs presented ‘auditable’ standards [33], [43], but their complexity in one would be difficult to operationalise [NICE] (http://www.nice.org.uk/guidance/qs35) and only one criterion is presented in the other CPG [WHO]. Our review suggests that where there is consistency between CPGs, there is the potential for standardisation of both: definitions that will support research efforts [48], [47], and quality of care criteria, particularly if the between-CPG differences in quality of evidence/strength of recommendation can be resolved.

The AGREE II tool is the standard for assessing the quality of published CPGs [13]. However, it has never been shown to improve guideline uptake or implementation [8], and use of the AGREE II presents some difficulties. First, AGREE II lists many criteria and few CPGs in our review scored highly on some or most domains, which may reflect space limitations in the journal of publication, rather than guideline quality. Second, AGREE II scores do not reflect important usability issues, such as the length of the CPG document and appendices/evidence tables (extensive for the NICE guideline), number of formulated recommendations, and presentation of the grading of the evidence relative to the recommendation, or lack of assessment of the strength of the recommendation (absent from AGREE II), all of which must be considered when evaluating how easy guidelines would be to use clinically. Although we did not exclude any CPGs based on their AGREE II domain scores, the four CPGs deemed ‘not useful’ for busy clinical practice did receive the lowest scores [ASH, DGGG, ESC and SOMANZ].

### Strengths and limitations

The strengths of our review include: a comprehensive literature search by an information technology specialist, inclusion of CPGs published in the last 10 years and in any of four languages, and the systematic summary of the diagnostic criteria and stated recommendations for the prevention of pre-eclampsia and treatment of the HDPs, incorporating quality of evidence and strength of recommendations. Although it is recommended that guidelines be updated every 3–5 years [49]–[51], the time consuming work of developing CPGs could mean that some guidelines are not updated as frequently as recommended and in our review, limiting to publication within the last 5 years would have excluded 4/13 CPGs.

Our review has limitations. A potential selection bias exists in the fact that only CPGs written in English, German, French and Dutch were included; however, we excluded only two CPGs (in Spanish [28], [29]) for this reason; the French CPG excluded was because it was available only by purchase for a significant sum [26]. The CPGs were assessed by two appraisers; this approach meets the minimum number of appraisers advised by AGREE II, but some authors have used more. As our focus was on the clinician, we did not extract and compare information available only from guideline text, some of which runs to 288 pages (even without appendices or evidence tables) [NICE] and none of which has associated ‘strength of recommendation’ that would aid the reader in deciding whether to comply or not. Finally, discrepancies in grading of evidence between different systems for the same recommendation were noted.

## Conclusions

The existing CPGs that inform care for women with a HDP have areas of consistency with which clinicians and researchers can work to develop auditable standards, and areas of inconsistency that should be addressed by future research.

## Supporting Information

Table S1 **Diagnosis and classification of HDPs.**
(DOC)

Table S2 **Definitions of Preeclampsia and severe Preeclampsia.**
(DOCX)

Table S3 **Recommendations concerning the Prediction of HDP.**
(DOC)

Table S4 **Recommendations concerning the Prevention of HDP.**
(DOC)

Table S5 **Recommendations concerning the Treatment of HDP.**
(DOC)

Appendix S1 **Search strategy.**
(DOC)

Checklist S1 **PRISMA checklist.**
(DOC)
