# Supplementary material for: Hypertensive Disorders of Pregnancy: A Systematic Review of International Clinical Practice Guidelines
Source: PLoS One. 2014 Dec 1;9(12):e113715. doi: 10.1371/journal.pone.0113715 (PMC4249974; doi:10.1371/journal.pone.0113715)
Supplement: Table S1 — Diagnosis and classification of HDPs. (DOC) [file pone.0113715.s001.doc]

**Table S1: Diagnosis and classification of the hypertensive disorders of pregnancy**

|  | **PRECOG^34^**  **2005** | **PRECOG II^35^**  **2009** | **QLD^38,39^**  **2010** | **NICE^33^**  **2010** | **WHO^43^**  **2011** | **NVOG^40^**  **2011** | **AOM^32^**  **2012** | **ACOG^36^**  **2013** | **SOGC^30,31^**  **2014** |
| --- | --- | --- | --- | --- | --- | --- | --- | --- | --- |
| **Pre-existing (chronic) hypertension** | dBP ≥90mmHg before pregnancy or at booking before 20 wks | dBP ≥90 mmHg before pregnancy or at booking before 20 wks | (specify essential without known cause)  BP > 140/90 mmHg before pregnancy or 20 wks or if woman taking antihypertensive(s) when she conceives | “Hypertension” at booking or before 20 wks or if woman taking antihypertensives when referred to maternity services. |  | BP ≥140/90 mmHg before pregnancy or 20 wks. | Hypertension before pregnancy or 20 wks | Hypertension (≥140/90) before pregnancy or 20 wks | Hypertension (≥140/90) before pregnancy or 20 wks |
| ***With co-morbid conditions*** |  |  | “Secondary” causes are listed |  |  |  | Co-morbid conditions are listed and some include some secondary causes (e.g., CKD) |  | Co-morbid conditions are listed and some include some secondary causes (e.g., CKD) |
| ***Superimposed PET*** | New features of PET (includes women with pre-existing proteinuria) | New features of PET | New systemic features of PET after 20 wks |  |  | Symptoms of PET after 20 wks | One/more at ≥ 20 wks: resistant hypertension or new or worsening proteinuria or one or more other adverse conditions | “More likely” when:  New proteiniuria after 20 wks  Sudden, substantial, and sustained increase in proteinuria  AND  (1) sudden increase in BP or need to increase antihypertensive dose;  sudden signs and symptoms of PET, such as  (2) abnormal liver enzymes;  (3) platelet count <100,000 cells/mm3;  (4) PET symptoms such as right upper quadrant pain and severe headaches;  (5) pulmonary congestion or edema;  (6) renal insufficiency (creatinine level doubling or rising to ≥1.1 mg/dL (97.2μM) in women without other renal disease | One/more at ≥20 wks:  Resistant hypertension, *or*  New or worsening proteinuria, *or*  One/more adverse condition(s), *or*  One/more severe complication(s) |
| ***Includes women with pre-existing proteinuria*** | **√** |  |  |  |  |  | **√** | **√** | **√** |
| ***Superimposed PET without severe features*** |  |  |  |  |  |  |  | Without organ system dysfunction #2-6 above (i.e., only hypertension and proteinuria) |  |
| ***Superimposed PET with severe features*** |  |  |  |  |  |  |  | With one/more organ dysfunctions (#2-6 above) |  |
| **Resistant hypertension** |  |  |  |  |  |  |  |  | Need for three antihypertensives for BP control at ≥20 wks |
| **Gestational or ‘new’ hypertension** | New hypertension at ≥20 wks | New hypertension at ≥20 wks | New hypertension at >20 wks, without features of PET, with normal BP by 12 wks postpartum | New hypertension at >20 wks without proteinuria |  | New sBP ≥ 140 mmHg and/or dBP ≥90 mmHg (KV) at >20 wks, measured twice, with normal BP at 12 wks postpartum | New hypertension at ≥20 wks | New hypertension at >20 wks without proteinuria, with normal BP “postpartum” | New hypertension at ≥20 wks |
| ***With co-morbid conditions*** |  |  |  |  |  |  | Co-morbid conditions are listed and some include some secondary causes (e.g., CKD) |  | Co-morbid conditions are listed and some include some secondary causes (e.g., CKD) |
| ***With evidence of pre-eclampsia*** |  |  |  |  |  |  | New proteinuria *or* one or more of the other adverse conditions (see table 3). |  | New proteinuria or one/more of: adverse condition(s)¥ *or* severe complication(s)¥ |
| **Pre-eclampsia** | Gestational hypertension and quantified proteinuria that resolves after delivery. | Gestational hypertension and proteinuria that resolves after delivery | Gestational hypertension(confirmed twice) and proteinuiria or one/more of: renal involvement (creat ≥ 90 µmol/L or oliguria), haematological involvement (thrombocytopenia, haemolysis, DIC), liver involvement ( raised transaminases, severe epigastric or RUQ pain), neurological involvement (severe headache, persistent visual disturbances of photopsia, scotomata, or cortical blindness, retinal vasospasm, hyperreflexia with sustained clonus, convulsions (eclampsia), stroke, pulmonary oedema, IUGR, placental abruption | Gestational hypertension and proteinuria | Gestational hypertension and proteinuria (>0.3g/24hr) | Gestational hypertension and proteinuria (>0.3g/24hr)  Also defines mild pre-eclampsia | Hypertension and proteinuria or one/more of signs and symptoms associated with end-organ dysfunction | Gestational hypertension and new proteinuria or one/more of: thrombocytopenia (<100,000 platelets/mL), impaired liver function (elevated blood levels of live transaminases to 2x normal), new development of renal insufficiency (creat >1.1mg/dL or a doubling of serum creat in the absence of other renal disease), pulmonary edema, or cerebral or visual disturbances | Gestational hypertension and new proteinuria or one/more of: adverse condition(s)¥ *or severe* complication(s)¥ |
| ***Eclampsia*** |  |  | With PET, one/more seizures | With PET, a convulsive condition | With PET, generalized seizures not attributable to other causes |  | With PET, new onset of convulsions | With PET, new onset grand mal seizures |  |
| ***Severe pre-eclampsia*** |  |  | One/more of: platelet count <100,00 x 10^9^/L, elevated transaminases, microangiopathic haemolytic anaemia with fragments/schistocytes on blood film (essentially HELLP syndrome) | Severe hypertension and/or symptoms, and/or biochemical and/or haematological impairment | One/more of: severe hypertension, heavy proteinuria, and substantial maternal organ dysfunction  Onset at <32-34 wks and fetal morbidity are used in some parts of the world | Severe hypertension or PET symptoms (headache, epigastric pain, nausea, malaise), or proteinuria > 5g/24 hr | PET with onset at <34 wks, with heavy proteinuria (> 0.3-0.5g/24hr) or with one/more adverse conditions | (p32) **”…consideration of pre-eclampsia as mild should be avoided.” | PET with one/more severe complications‡. |
| ***HELLP syndrome*** |  |  | HELLP spelled out  Highlighted as variant of severe pre-eclampsia | HELLP spelled out |  |  |  | HELLP spelled out  Highlighted as a pre-eclamptic subtype |  |
| **Other ‘hypertensive effects’** |  |  |  |  |  |  |  |  |  |
| **Transient hypertensive effect** |  |  |  |  |  |  |  |  | Elevated BP may be due to environmental stimuli or the, pain of labour, for example. |
| **White-coat effect** |  |  | BP that is elevated in a clinical setting but normal in a non-clinical setting by (24 hr) ABPM or HBPM using an appropriately validated device |  |  |  |  |  | BP that is elevated in a clinical setting but normal in a non-clinical setting (<135/85 mmHg) by ABPM or HBPM |
| **Masked hypertensive effect** |  |  |  |  |  |  |  |  | BP that is normal in the clinical setting but elevated in a non-clinical setting (≥135/85 mmHg) by ABPM or HBPM |
| **Hypertension (sBP and/or dBP)** | dBP ≥90 mmHg | dBP ≥90 mmHg | sBP ≥140 mmHg  and/or  dBP ≥90 mmHg | dBP ≥90 mmHg (on two occasions,>4 hours appart)  or  dBP >110 mmHg (measured once) | - | sBP ≥140 mmHg  and/or  dBP ≥90 mmHg | dBP ≥90 mmHg | sBP ≥140 mmHg  or  dBP ≥90 mmHg | sBP ≥140 mmHg  and/or  dBP ≥90 mmHg  (based on average ≥2 measurements, taken ≥15 min apart, using the same arm) |
| ***Mild*** |  |  |  | sBP 140-149 mmHg  dBP 90-99 mmHg |  |  |  | sBP 140-159 mmHg  or  dBP 90-109 mmHg |  |
| ***Moderate*** |  |  |  | sBP 150-159 mmHg  dBP 100-109 mmHg |  | sBP 140-159 mmHg  or  dBP 90-109 mmHg |  |  |  |
| ***Severe*** |  |  | ≥160/ and/or 110 mmHg | ≥160/110 mmHg |  | ≥160/or 110 mmHg | ≥160/or 110 mmHg | ≥160/or 110 mmHg    (as greater than mild) | ≥160/or 110 mmHg    (based on average ≥2 measurements, taken ≥15 min apart, using the same arm) |
| **Late postpartum hypertenison** |  |  |  |  |  |  |  | Hypertension (usually mild) that develops 2wks to-6mos postpartum, usually normalizing by the end of the first year. |  |

ABPM (ambulatory blood pressure monitoring), ACOG (American College of Obstetricians and Gynecologists), AOM (Association of Ontario Midwives), BP (blood pressure), CKD (chronic kidney disease), Creat (creatinine), dBP (diastolic blood pressure), DIC (disseminated intravascular coagulation), HBPM (home blood pressure monitoring), HELLP syndrome (Haemolysis, Elevated Liver enzymes and Low Platelet count syndrome), NICE (National Institute for Health and Clinical Excellence ), NVOG (Nederlandse Vereniging voor Obstetrie en Gynaecologie), PET (pre-eclampsia), PRECOG (pre-eclampsia community guideline), QLD (Queensland Maternity and Neonatal Clinical Guidelines Program), RUQ (right upper quadrant), sBP (systolic blood pressure), SOGC (Society of Obstetricians and Gynaecologists of Canada), WHO (World Health Organisation)
