# Supplementary material for: Hypertensive Disorders of Pregnancy: A Systematic Review of International Clinical Practice Guidelines
Source: PLoS One. 2014 Dec 1;9(12):e113715. doi: 10.1371/journal.pone.0113715 (PMC4249974; doi:10.1371/journal.pone.0113715)
Supplement: Table S2 — Definitions of Preeclampsia and severe Preeclampsia. (DOCX) [file pone.0113715.s002.docx]

**Table S2: Definitions of pre-eclampsia and severe pre-eclampsia ***

|  | Define **pre-eclampsia** in association with hypertension | | | | | | | | | Define **SEVERE pre-eclampsia** | | | | | | | | | Notes |
| --- | --- | --- | --- | --- | --- | --- | --- | --- | --- | --- | --- | --- | --- | --- | --- | --- | --- | --- | --- |
|  | **PRECOG^34^**  **2005** | **PRECOG II^35^**  **2009** | **QLD^38,39^**  **2010** | **NICE^33^**  **2010** | **WHO^43^**  **2011** | **NVOG^40^**  **2011** | **AOM^32^**  **2012** | **ACOG^36^**  **2013** | **SOGC^30,31^**  **2014** | **PRECOG^34^**  **2005** | **PRECOG II^35^**  **2009** | **QLD^38,39^**  **2010** | **NICE^33^**  **2010 †** | **WHO^43^**  **2011 **** | **NVOG^40^**  **2011** | **AOM^32^**  **2012** | **ACOG^36^**  **2013 ***** | **SOGC^30,31^**  **2014** |  |
| **Proteinuria** | **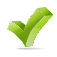** | **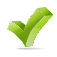** | **^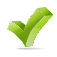1^** | **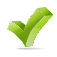** | **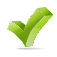** | **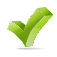** | **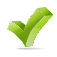^1^** | **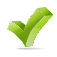^1^** | **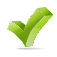^1^** |  |  |  |  |  |  |  |  |  | 1. not mandatory. In absence of proteinuria, one or more of 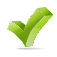 |
| Heavy proteinuria |  |  |  |  |  |  |  |  |  |  |  |  |  | 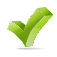 | **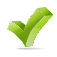** | **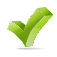** |  |  |  |
| **Proteinuria is not mandatory – pne/more other manifestations sufficient** |  |  | **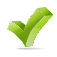** |  |  |  | **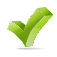** | **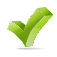** | **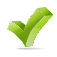** |  |  |  |  |  |  |  |  |  |  |
| **Gestational age at onset** <34 wk |  |  |  |  |  |  |  |  |  |  |  |  |  | 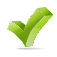^1^ |  | 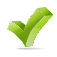 |  | ^2^ | 1. <32-34wks  2. mentioned in text as risk factor for poor outcome |
| **Maternal symptoms** |  |  |  |  |  |  |  |  |  |  |  |  |  |  |  |  |  |  |  |
| Headache/visual symptoms |  |  | 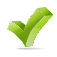 |  |  |  | 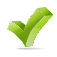 | 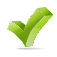^1^ | 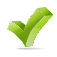 |  |  |  | 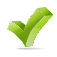 |  | 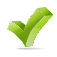 | 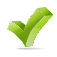 | 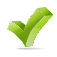^2^ |  | 1. Cerebral or visual disturbances  2. Cerebral or visual disturbances (with proteinuria) |
| Chest pain/dyspnoea |  |  |  |  |  |  | 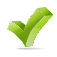 |  | 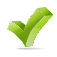 |  |  |  | 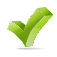 |  |  | 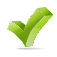 |  |  |  |
| Nausea/vomiting |  |  |  |  |  |  | 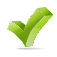 |  | 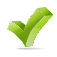 |  |  |  | 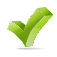 |  | 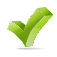 | 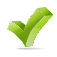 |  |  |  |
| Right upper quadrant/epigastric pain |  |  | 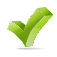 |  |  |  | 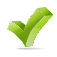 |  | 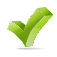 |  |  |  | 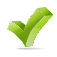 |  | 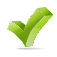 | 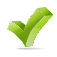 | 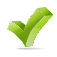^1^ |  | 1. Severe, persistent, unresponsive to medication, not otherwise explained (with proteinuria) |
| **Maternal signs** |  |  |  |  |  |  |  |  |  |  |  |  |  |  |  |  |  |  |  |
| **Cardiac/ cardiovascular** |  |  |  |  |  |  |  |  |  |  |  |  |  |  |  |  |  |  |  |
| **Severe hypertension** |  |  |  |  |  |  | **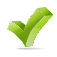** |  | **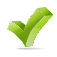** |  |  |  | **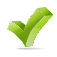** | **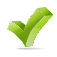** | 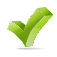 | 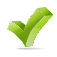 | 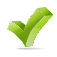 |  |  |
| Uncontrolled severe hypertension |  |  |  |  |  |  |  |  |  |  |  |  | (**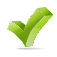**) | (**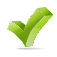**) |  |  |  | **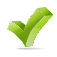** |  |
| Positive inotropic support |  |  |  |  |  |  |  |  |  |  |  |  |  | (**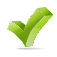**) |  |  |  | **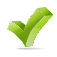** |  |
| Myocardial ischemia/infarction |  |  |  |  |  |  |  |  |  |  |  |  |  | (**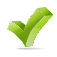**) |  |  |  | **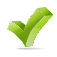** |  |
| **Neurologic** |  |  |  |  |  |  |  |  |  |  |  |  |  |  |  |  |  |  |  |
| Eclampsia |  |  | **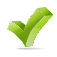** |  |  |  | **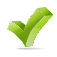** |  |  |  |  |  |  | (**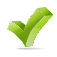**) |  | **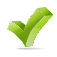** |  | **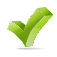** |  |
| PRES |  |  |  |  |  |  |  |  |  |  |  |  |  | (**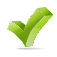**) |  |  |  | **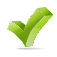** |  |
| Cortical blindness or retinal detachment |  |  | **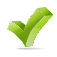** |  |  |  |  |  |  |  |  |  |  | (**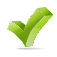**) |  | **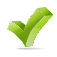** |  | **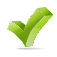** |  |
| Glasgow coma scale <13 |  |  |  |  |  |  |  |  |  |  |  |  |  | (**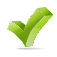**) |  |  |  | **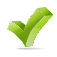** |  |
| Stroke, TIA or RIND |  |  | **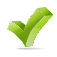** |  |  |  |  |  |  |  |  |  |  | (**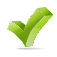**) |  |  |  | **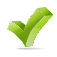** |  |
| Hypereflexia (with clonus) |  |  | **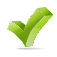** |  |  |  |  |  |  |  |  |  |  |  |  |  |  |  |  |
| **Pulmonary** |  |  |  |  |  |  |  |  |  |  |  |  |  |  |  |  |  |  |  |
| Oxygen saturation <97% |  |  |  |  |  |  |  |  | 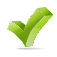 |  |  |  |  |  |  |  |  |  |  |
| Oxygen saturation <90% |  |  |  |  |  |  |  |  |  |  |  |  |  | (**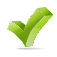**) |  |  |  | **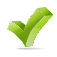** |  |
| Pulmonary edema |  |  | **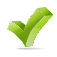** |  |  |  | **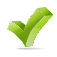** | **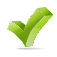** |  |  |  |  |  | (**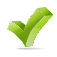**) |  | **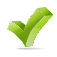** | **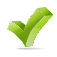** ^1^ | **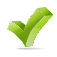** | 1. with proteinuria |
| Need for ≥50% oxygen for >1hr |  |  |  |  |  |  |  |  |  |  |  |  |  | (**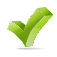**) |  |  |  | **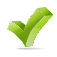** |  |
| Intubation (other than for Caesarean delivery), |  |  |  |  |  |  |  |  |  |  |  |  |  | (**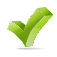**) |  |  |  | **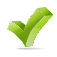** |  |
| **Renal** |  |  |  |  |  |  |  |  |  |  |  |  |  |  |  |  |  |  |  |
| Oliguria |  |  | **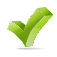** |  |  |  |  |  |  |  |  |  |  |  |  |  |  |  |  |
| **Abnormal maternal laboratory tests** |  |  |  |  |  |  |  |  |  |  |  |  |  |  |  |  |  |  |  |
| **Haematology/coagulation** |  |  |  |  |  |  |  |  |  |  |  | 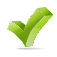^1^ |  |  |  |  |  |  | 1.“ microangiopathic haemolytic anaemia” |
| Elevated WBC count |  |  |  |  |  |  |  |  | 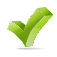 |  |  |  |  |  |  |  |  |  |  |
| Platelet count decreased but ≥50x10^9^/L |  |  |  |  |  |  |  |  | 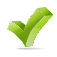 |  |  |  |  |  |  |  |  |  |  |
| Platelet count decreased but <50x10^9^/L |  |  | 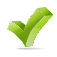^1^ |  |  |  | 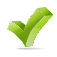^2^ | 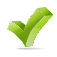^2^ |  |  |  | 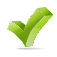^2^ | (**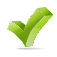**) | (**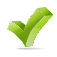**) |  | 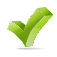^2^ | 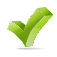^3^ |  | 1. thrombocytopenia  2. <100,000/mL  3. <100,000/mL with proteinuria |
| Elevated INR or aPTT |  |  | ^1^ |  |  |  |  |  |  |  |  |  |  |  |  |  |  |  | 1. hemolysis and DIC |
| **Renal** |  |  |  |  |  |  |  |  |  |  |  |  |  |  |  |  |  |  |  |
| Elevated serum uric acid |  |  |  |  |  |  |  |  |  |  |  |  |  |  |  |  |  |  |  |
| Elevated serum creatinine |  |  |  |  |  |  |  |  |  |  |  |  |  |  |  |  |  |  |  |
| Acute kidney injury (creatinine >150μM with no prior renal disease) |  |  |  |  |  |  |  |  |  |  |  |  | () | () |  |  | ^1, 2^ |  | 1. Progressive renal insufficiency (serum creatinine >1.1mg/dL or a doubling of serum creatinine concentration in absence of other renal disease)  2. with proteinuria |
| New indication for dialysis |  |  |  |  |  |  |  |  |  |  |  |  | () | () |  |  |  |  |  |
| **Hepatic** |  |  |  |  |  |  |  |  |  |  |  |  |  |  |  |  |  |  |  |
| Elevated serum AST, ALT, LDH or bilirubin |  |  |  |  |  |  |  | 1 |  |  |  |  |  |  |  |  | ^1, 2^ |  | 1. Twice normal  2. With proteinuria |
| Hepatic dysfunction (INR >2 in absence of DIC or warfarin) |  |  |  |  |  |  |  |  |  |  |  |  | () | () |  |  |  |  |  |
| Low plasma albumin |  |  |  |  |  |  |  |  |  |  |  |  |  |  |  |  |  |  |  |
| Hepatic haematoma or rupture |  |  |  |  |  |  |  |  |  |  |  |  | () | () |  |  |  |  |  |
| **Fetoplacental manifestations** |  |  |  |  |  |  |  |  |  |  |  |  |  |  |  |  |  |  |  |
| Non-reassuring FHR |  |  |  |  |  |  |  |  |  |  |  |  |  |  |  |  |  |  |  |
| IUGR |  |  |  |  |  |  |  |  |  |  |  |  |  |  |  |  | **1** |  | 1. Not included (as IUGR with PET managed the same way as IUGR w/o PET) |
| Oligohydramnios |  |  |  |  |  |  |  |  |  |  |  |  |  |  |  |  |  |  |  |
| Absent/reversed end-diastolic flow by Doppler velocimetry |  |  |  |  |  |  |  |  |  |  |  |  |  |  |  |  |  |  |  |
| Abruption without evidence of maternal or fetal compromise |  |  | () |  |  |  | () |  |  |  |  |  |  |  |  | () |  |  |  |
| Abruption with evidence of maternal or fetal compromise |  |  | () |  |  |  | () |  |  |  |  |  |  | () |  | () |  |  |  |
| Reverse ductus venosus A wave |  |  |  |  |  |  |  |  |  |  |  |  |  |  |  |  |  |  |  |
| Stillbirth |  |  |  |  |  |  |  |  |  |  |  |  |  |  |  |  |  |  |  |
| **Interventions** |  |  |  |  |  |  |  |  |  |  |  |  |  |  |  |  |  |  |  |
| Transfusion of any blood product |  |  |  |  |  |  |  |  |  |  |  |  |  |  |  |  |  |  |  |

ACOG (American College of Obstetricians and Gynecologists), AOM (Association of Ontario Midwives), aPTT (activated partial thromboplastic time), ASH (American Society of Hypertension), AST (aspartate aminotransferase), ALT (alanine aminotransferase), FHR (fetal heart rate), INR (international normalised ratio), IUGR (intrauterine fetal growth restriction), LDH (lactate dehydrogenase), NICE (National Institute for Health and Clinical Excellence), NVOG (Nederlandse Vereniging voor Obstetrie en Gynaecologie), PRECOG (pre-eclampsia community guideline), PRES (posterior reversible encephalopathy syndrome), QLD (Queensland Maternity and Neonatal Clinical Guidelines Program), RIND (reversible ischaemic neurological deficit), SOGC (Society of Obstetricians and Gynaecologists of Canada), TIA (transient ischaemic attack), WBC (white blood cell count), WHO (World Health Organization)

*** A checkmark indicates that the diagnostic criterion was listed by the guideline. A checkmark in brackets indicates that although not listed specifically, the criterion could reasonably be interpreted as being part of the definition in the relevant guideline.**

**†** The NICE 2010 guidelines include “symptoms, and/or biochemical and/or haematological impairement” as part of the definition of severe pre-eclampsia. It is assumed that those complications indicated by () would meet this definition.

** The WHO 2011 guidelines include “substantial maternal end-organ dysfunction” as part of the definition of severe pre-eclampsia. It is assumed that those complications indicated by () would meet this definition. “Fetal morbidity” also required interpretation.

*** Pre-eclampsia with severe feature.
