# Supplementary material for: Hypertensive Disorders of Pregnancy: A Systematic Review of International Clinical Practice Guidelines
Source: PLoS One. 2014 Dec 1;9(12):e113715. doi: 10.1371/journal.pone.0113715 (PMC4249974; doi:10.1371/journal.pone.0113715)
Supplement: Table S3 — Recommendations concerning the Prediction of HDP. (DOC) [file pone.0113715.s003.doc]

**Table S3: Prediction of pre-eclampsia ***

|  | **PRECOG^34^ 2005** | **NICE^33^ 2010** | **WHO^43^ 2011** | **AOM^32^**  **2012** | **ACOG^36^ 2013** | **SOGC^30,31^ 2014** |
| --- | --- | --- | --- | --- | --- | --- |
| **PREDICTION** |  |  |  |  |  |  |
| **Risk assessment** |  |  |  |  |  |  |
| ***Methods*** |  |  |  | Offer screening for PET by clinical assessment in early pregnancy, and decide whether or not to undertake preventive measures  (IIIB)  (IIIA/B) | Offer screening for PET risk by clinical assessment  (Moderate, Strong) | Offer screening for PET risk by clinical assessment in early pregnancy (II-2C / Low, Strong)  Do NOT offer routine screening using biomarkers or Doppler ultrasound velocimetry of uteroplacental circulation, even in women at increased risk of PET  (II-2C / Very low,Weak) |
| ***Clinical risk markers for PET*** | History of previous preeclampsia  Multiple pregnancy  Antiphospholipid antibodies  Significant proteinuria at booking or pre-existing renal disease  Pre-existing diabetes mellitus  Pre-existing hypertension  (D, GPP)  (B/C)  Two/more risk markers of:  First pregnancy  ≥10yr since last baby  Age ≥40yr  BMI ≥35  Family history of pre-eclampsia (mother/sister),  Booking diastolic BP ≥80mmHg  (B/C) | “High” risk markers:  HDP in prior pregnancy  Autoimmune disease (e.g., SLE)  Antiphospholipid syndrome  Renal disease  Pre-existing diabetes mellitus  Pre-existing hypertension  “Moderate” risk factors:  Multiple pregnancy  First pregnancy  Age ≥40 yr  >10yr since lst baby  BMI ≥35 kg/m² at first visit  Family history of PET  ≥10 yr since last baby | Listed risk factors:  Obesity, chronic hypertension, DM, nulliparity, adolescent pregnancy, conditions leading to hyperplacentation and large placentas (eg. Twin pregnancy) | Listed risk markers:  Presence of antiphospholipid antibodies, previous PET, pre-existing DM, multiple pregrancy, nulliparity, family history of PET, raised pre-pregnancy BMI, maternal age ≥ 40  Less well established risk factors:  Length of exposure to single partner’s sperm, previous fathering of a preeclamptic pregnancy, inter-pregnancy or interbirth interval, use of donor oocyte. | Listed risk factors:  First degree relative with history of PET, PET in previous PET, multiple gestation, maternal age ≥ 40, DM, obesity, preexisting hypertension  (Comment from text: “most cases of PET occur in healthy nulliparous women with no other obvious risks”) | History of previous preeclampsia  Multiple pregnancy  Antiphospholipid antibody syndrome  Significant proteinuria at booking or pre-existing renal disease  Pre-existing diabetes mellitus  Pre-existing hypertension  (II-2 B / Very low, Strong) |
| **Consultation with an obstetrician or an obstetric internist** |  |  |  | Offer “consultations” as indicated by midwifery governing body (IIIA/B) | Offer counselling and assessment to women with previous PET  (Low, Qualified) | Offer consultation with an obstetrician or obstetric internist to women with previous PET or another strong clinical risk marker for PET  (II-2 B / Very low, Strong) |
| **Frequency of antenatal care** | Offer women with no ‘Box 2’ risk markers for PET (low risk multiparous women), midwife or GP led community monitoring from 24 wks until delivery, “as per local protocols and NICE antenatal guideline for low risk multiparous women”  (B)  Offer women with one ‘Box 2’ but no ‘Box 3’ risk markers for PET, midwife or GP led community monitoring visits at minimum, every 3 wks from 24-32wks and every 2 wks from 32 wks to delivery  (B) |  |  |  |  |  |

ACOG (American College of Obstetricians and Gynecologists), AOM (Association of Ontario Midwives), BMI (body mass index), DM (diabetes mellitus), GP (general practitioner), GPP (good practice point), NICE (National Institute for Health and Clinical Excellence ), PET (pre-eclampsia), PRECOG (pre-eclampsia community guideline), WHO (World Health Organisation), SOGC (Society of Obstetricians and Gynaecologists of Canada)

* Refer to Tables 2a and 2b for definitions of both the quality of the evidence and the strength of the recommendations as listed by individual guidelines. Yellow highlighting refers to information found in the footnotes of tables or in the text but linked with recommendations for easy identification.
