# Supplementary material for: Hypertensive Disorders of Pregnancy: A Systematic Review of International Clinical Practice Guidelines
Source: PLoS One. 2014 Dec 1;9(12):e113715. doi: 10.1371/journal.pone.0113715 (PMC4249974; doi:10.1371/journal.pone.0113715)
Supplement: Table S4 — Recommendations concerning the Prevention of HDP. (DOC) [file pone.0113715.s004.doc]

**Table S4: Prevention of pre-eclampsia ***

|  | **NICE^33^ 2010** | **WHO^43^ 2011** | **NVOG^40^ 2011** | **AOM^32^ 2012** | **ACOG^36^ 2013** | **SOGC^30,31^ 2014** |
| --- | --- | --- | --- | --- | --- | --- |
| **PREVENTING PREECLAMPSIA** |  |  |  |  |  |  |
| **General strategies to prevent adverse outcomes in pregnancy** | Rest and exercise as for all pregnant women |  | Rest and exercise as for all pregnant women |  |  | Abstention from alcohol for prevention of fetal alcohol effects  (II-2E / Low, Strong)    Exercise for maintenance of fitness  (I-A / Moderate, Strong)  Periconceptual use of a folate-containing multivitamin for prevention of neural tube defects  (I-A / Moderate, Strong)  Smoking cessation for prevention of low birthweight and preterm birth  (I-E / High, Strong)  Heparin to prevent VTE  (I-B / Moderate, Weak) |
| **Women at low risk** |  |  |  |  |  |  |
| **Recommended** |  | Calcium supplementation (1.5-2g/d) for women in areas with low dietary calcium intake  (Moderate, Strong) |  |  |  | Calcium supplementation (≥1g/d) for women with low dietary calcium intake (<600mg/d)  (I-A / High, Strong)  Periconceptual and ongoing use of a folate-containing multivitamin “may be useful”  (I-B / Low, Weak)  Exercise “may be useful”  (II-2B / Very low, Weak) |
| **NOT recommended for PET prevention** |  |  |  |  |  | Prostaglandin precursors  (I-C / Low, Weak)  Magnesium  (I-C / Low, Weak)  Zinc  (I-C / Low, Weak) |
| **Not recommended** | Dietary salt restriction  Vitamins C and E  Diuretics  Nitric oxide donors  Progesterone  Magnesium  Folic acid  Fish oils or algal oils  Garlic  Low molecular weight heparin | Dietary salt restriction (Moderate, Weak)  Vitamins C and E  (High, Strong)  Diuretics  (Low, Strong)  Vitamin D  (Very low, Strong) |  |  | Dietary salt restriction  (Low, Qualified)  Bed rest or the restriction of other physical activity  (Low, Qualified)  Vitamins C or E (High, Strong) | Dietary salt restriction  (I-D / Moderate, Strong)  Calorie restriction for overweight women  (I-D / Moderate, Strong)  Low-dose aspirin (I-E / Moderate, Weak)  Vitamins C and E (I-E / High, Strong)  Thiazide diuretics  (I-E / Moderate, Strong) |
| **Insufficient evidence** |  |  |  |  |  | Heart-healthy diet  (II-2L / Very low, Weak)  Workload or stress reduction  (II-2L / Very low, Weak)  Supplementation with iron with/without folate  (I-L / Low, Weak)  Vitamin D  (I-L / Very low, Weak)  Pyridoxine  (I-L / Low, Weak)  Food rich in flavanoids  (I-L / Very low, Weak) |
| **Women at increased risk** | One/more “high” risk markers  Two/more “moderate” risk markers | “women were regarded as being at high risk if they were normotensive or had chronic hypertension in addition to one or more of the following risk factors: previous severe PET; DM; chronic htn; renal disease; or autoimmune disease.” |  | (due to presence of chronic htn, DM, kidney disease, autoimmune disorder or previous severe PET) | Prior early-onset PET and preterm delivery at < 34 0/7 wks  Prior recurrent PET | One or more risk of risk factors listed above |
| **Recommended** |  |  |  |  |  |  |
| ***ASA*** | Low dose ASA  Dose of 75 mg/d  Taken from 12 wks  Taken until birth | Low dose ASA  (Moderate, Strong)  Dose of 75mg  (Moderate, Strong)    Taken from before 20 (+0) wks  (Low, Weak) |  | Low dose ASA  (IA)  Dose of 81 mg/d  (IA)  Taken from time when increased risk of PET is identified, ideally before 16 wks  (IA)  Taken until delivery  (IA) | Low dose ASA  (Moderate, Qualified)  Dose of 60-80mg/d  (Moderate, Qualified)  Taken from late in first trimester  (Moderate, Qualified) | Low dose ASA  (I-A / High, Strong)  Dose of 75–162 mg/d  (III-B / Very low, Weak)  Taken from at bedtime, after diagnosis of pregnancy but before 16 wks  (I-B / Moderate, Strong)  Taken until delivery  (I-C / Very low, Weak) |
| ***Calcium*** |  | Calcium supplementation (of 1.5-2 g/d) in areas where dietary calcium intake is low  (Moderate, Strong) |  | Calcium supplementation or increased intake (of 1-2.5 g/d) in women  (IA/B) |  | Calcium supplementation (of at least 1 g/d) for women with low calcium intake  (I-A / High, Strong) |
| ***Other*** |  |  |  |  |  | L-arginine  (I-B / Moderate, Weak)  Increased rest at home in the third trimester  (I-C / Low, Weak)  Reduction of workload or stress  (III-C / Very low, Weak)  Prophylactic doses of LMWH may be discussed in women with previous placental complications (including PET) to prevent the recurrence of ‘severe’ or early-onset preeclampsia, preterm delivery, and/or SGA infants  (I-B / Moderate, Weak) |
| **NOT recommended** but may be useful for other pregnancy complications |  |  |  |  |  | Prostaglandin precursors  (I-B / Low, Weak)  Magnesium  (I-C / Low, Weak) |
| **Not recommended** | Dietary salt restriction  Vitamins C and E  Diuretics  Nitric oxide donors  Progesterone  Magnesium  Folic acid  Fish oils or algal oils  Garlic  Low molecular weight heparin | Dietary salt restriction (Moderate, Weak)  Rest at home  (Low, Weak)  Vitamins C and/or E  (High, Strong)  Diuretics,, particularly thiazides  (Low, Strong)  Vitamin D  (Very low, Strong) |  |  | Dietary salt restriction  (Low, Qualified)  Bed rest or restriction of physical activity  (Low, Qualified)  Vitamins C or E  (High, Strong) | Calorie restriction in overweight women  (I-D / Low, Weak)  Weight maintenance in obese women during pregnancy  (III-D / Very low, Weak)  Antihypertensive therapy  (I-D / Moderate, Strong)  Vitamins C and E (I-E / High, Strong) |
| **Insufficient evidence** |  |  |  |  |  | Heart-healthy diet  (III-L / Very low, Weak)  Exercise  (I-L / Very low, Weak)  Selenium  (I-L / Very low, Weak)  Garlic  (I-L / Very low, Weak)  Zinc  (III-L / Very low, Weak)  Pyridoxine  (III-L / Very low, Weak)  Iron (with or without folate)  (III-L / Very low, Weak)  Vitamin D  (III-L / Very low, Weak)  Multivitamins with/without micronutrients (III-L / Very low, Weak) |

ACOG (American College of Obstetricians and Gynecologists), AOM (Association of Ontario Midwives), ASA (aspirin), NICE (National Institute for Health and Clinical Excellence ), NVOG (Nederlandse Vereniging voor Obstetrie en Gynaecologie), SOGC (Society of Obstetricians and Gynaecologists of Canada), WHO (World Health Organisation)

* Refer to Tables 2a and 2b for definitions of both the quality of the evidence and the strength of the recommendations as listed by individual guidelines. Yellow highlighting refers to information found in the footnotes of tables or in the text but linked with recommendations for easy identification.
