# Supplementary material for: Hypertensive Disorders of Pregnancy: A Systematic Review of International Clinical Practice Guidelines
Source: PLoS One. 2014 Dec 1;9(12):e113715. doi: 10.1371/journal.pone.0113715 (PMC4249974; doi:10.1371/journal.pone.0113715)
Supplement: Table S5 — Recommendations concerning the Treatment of HDP. (DOC) [file pone.0113715.s005.doc]

**Table S5: Treatment of the hypertensive disorders of pregnancy ***

|  | **PRECOG II^35^**  **2009** | **QLD^38,38^**  **2010** | **NICE ^33^**  **2010** | **WHO^43^**  **2011** | **NVOG^40^**  **2011** | **AOM^32^**  **2012** | **ACOG^36^**  **2013** | **SOGC^30,31^**  **2014** |
| --- | --- | --- | --- | --- | --- | --- | --- | --- |
| **Dietary & lifestyle change** |  |  |  |  |  |  |  |  |
| **General comments** |  |  |  |  |  |  |  |  |
| **Dietary changes** |  |  | For women with chronic hypertension, ongoing salt restriction recommended |  | For women with chronic hypertension, ongoing salt restriction recommended |  | For women with chronic hypertension, extreme salt restriction NOT recommended (Low, qualified)  For women with chronic hypertension, weight loss NOT recommended  (Low, qualified) | For women with chronic hypertension, insufficient evidence to recommend ongoing salt restriction or  extreme (new) salt restriction  (III-L / Very low, Weak)  For women with chronic hypertension and obesity, insufficient evidence to recommend calorie restriction or heart healthy diet  (III-L / Very low, Weak) |
| **Exercise** |  |  |  |  |  |  | For women with chronic hypertension and BP that is controlled, ongoing (moderate) exercise recommended  (Low, qualified) | For women with any HDP, insufficient evidence to recommend  (III-L / Very low, Weak) |
| **Workload reduction** |  |  |  |  |  |  |  | For women with any HDP, insufficient evidence to recommend  (III-L / Very low, Weak) |
| **Stress reduction** |  |  |  |  |  |  |  | Stress reduction for any HDP – insufficient evidence to recommend  (III-L / Very low, Weak) |
| **Bedrest** |  |  | For women with GH, (any) NOT recommended | For women with any HDP, (strict) NOT recommended  (Low, Weak) |  |  | For women with GH or PET without severe features, (strict) NOT recommended  (Low, Qualified) | For women with GH, (In hospital vs unrestricted activity at home) may be useful  (I-B / Low, Weak)  For women with PET, (in hospital) NOT recommended (I-D / Moderate, Weak)  For women with chronic hypertension or any HDP out of hospital, Insufficient evidence to recommend  (III-C / Very low, Weak) |
| **Place of care** |  |  |  |  |  |  |  |  |
| **Transfer of care from midwifery** |  |  |  |  |  | PET  (IIIA) |  |  |
| **Assessment in secondary care setting by health care provider trained in HDP** |  |  | GH |  |  |  |  |  |
| **Hospital day unit or antepartum home care** |  |  |  |  |  |  |  | Consider for women with non-severe pre-existing hypertension, GH, or PET  (I-B / Moderate, Strong for day units and II-2B / Low, Strong for home care) |
| **Admit to hospital** | Women with any HDP and BP ≥170/110mmHg  (D)  (C with new proteinuria of ≥1+)  PET with proteinuria of ≥2+, or protein:creat ratio of ≥30  (C) | Women with any HDP and severe hypertension or severe PET (II-2B) | Women with GH |  |  |  |  | Women with any HDP and severe hypertension or “severe” PET  (II-2B / Low, Strong |
| **Refer to critical care setting** |  |  | Women with any HDP and severe hypertension or severe PET with specific end-organ dysfunction |  |  |  |  |  |
| **Antihypertensive therapy (antenatally or postnatally)** |  |  |  |  |  |  |  |  |
| **Antihypertensive therapy for severe Hypertension (defined)** |  | (≥160/110mmHg) | (≥160/110mmHg) |  | (≥160/110mmHg) | (≥160/110mmHg) | (≥160/110mmHg) | (≥160/110mmHg) |
| **Treatment recommended** |  | For women with any HDP, treat severe hypertension  (II-2B) | For women with any HDP, treat severe hypertension (immediately) during pregnancy or postpartum | For women with any HDP, treat severe hypertension  (Very Low, Strong) | For women with any HDP, treat severe hypertension |  | For women with any HDP, treat severe hypertension  (Moderate, Strong) | For women with any HDP, treat severe hypertension  (IA / Low, Strong) |
| **Target BP level** (level at which treatment may be unchanged; level above which treatment should be started; below which treatment should be decreased if on antihypertensive therapy) |  | For women with any HDP, goal of ≤160/100mmHg | For women with any HDP (in critical care), goal of <150/80-100 mmHg is recommended |  |  |  | For women with chronic hypertension, goal of <160/105mmHg is commended  (Moderate, Strong)  For women with PET, goal of <160/110  (Moderate, Stong) | For women with any HDP, goal of <160/110 mmHg is recomended  (I-A / Low, Strong) |
| **Initial antihypertensive therapy/first choice** |  | Initial anti-hypertensive therapy can be with one of a variety of antihypertensive drugs.  (GPP) | Labetalol (oral or iv, hydralazine (iv) or nifedipine (oral) are recommended for women in a critical care setting  Consider administration of up to 500ml of crystalloid before or with the first dose of hydralazine iv | Should be based on clinician’s experience, cost and local availability  (Very low, Weak) | Methyldopa, labetalol and nifedipine |  |  | Labetalol (iv), hydralazine (iv) or nifedipine (oral capsules) recommended  (all I-A / High, Strong)  Nifedipine and MgSO_4_ can be used contemporaneously  (II-2B / Moderate, Weak) |
| **Alternative antihypertensives** |  |  |  |  |  |  |  | Alternatives are **nitroglycerin** (iv) **methyldopa (oral)**, **labetalol (oral)**, clonidine (oral), or captopril (oral) only postpartum  (I-B / Moderate, Weak, for nitroglycerin, I-B / Very low, Weak for methyldopa, and I-B / Moderate, Weak for labetalol)  (III-B / Low, Weak for clonidine and captopril)  Sodium nitroprusside recommended for refractory hypertension  (III-B / Low, Weak) |
| **Antihypertensives NOT to use** |  |  |  |  | ACE inhibitors, ARBs and direct renin inhibitors during pregnancy |  |  | MgSO_4_ as an antihypertensive  (I-E / High, Strong) |
| **Other considerations** |  |  | For women with PET, consider side-effect profiles if giving treatment other than labetalol  For women with severe hypertension treated in critical care setting, monitor response to treatment , ensure BP falls, identify adverse effects, and modify treatment according to response |  |  |  |  | FHR monitoring (until stable BP) recommended  (III-I / Very low, Weak) |
| **For Non-Severe Hypertension** |  |  |  |  |  |  |  |  |
| **Target BP level** (level at which treatment may be unchanged; level above which treatment should be started or increased; level below which any antihypertensive therapy should be decreased) |  |  | For women with uncomplicated chronic hypertension, goal of <150/100 mmHg (without lowering dBP to <80mmHg) is recommended  For women with chronic hypertension and target organ damage, goal of  <140/90 mmHg recommended |  | For any HDP, goal of <160/110mmHg is recommended |  | For women with uncomplicated chronic hypertension, goal of 120-159/80-104mmHg is recommended (Low, Qualified) for upper limit  (Low, Qualified) for lower limit  For women with mild GH or PET, goal of <160/110mmHg is recommended  (Moderate, Qualified) | For any HDP, goal of 130–155/80–105 mmHg is recommended  (I-B / Low, Weak)  For women with any HDP and a co-morbid condition(s), goal of <140/90mmHg is recommended  (III-C / Very low, Weak) |
| **Antihypertensives to use** |  |  | For women with chronic hypertension, choose an agent(s) based on preexisting treatment, side-effect profiles and teratogenicity  For women with GH, offer antihypertensive medication (other than labetalol) ONLY after considering side effect profiles.  Alternatives include methyldopa† and nifedipine. |  | For women with any HDP, methyldopa, labetalol, and nifedipine recommended as agents of first choice |  | For women with chronic hypertension, methyldopa, labetalol, and nifedipine recommended as agents of first choice  (Moderate, Strong) | For women with any HDP, the choice of antihypertensive agent should be based on patient characteristics, contraindications and physician and patient preference  (III-C / Very low, Weak)  For women with any HDP, methyldopa, labetalol, nifedipine, other beta-blockers, or other calcium channel blockers are reasonable as agents of first choice  (I-A / High, Strong for all but other beta-blockers which were I-B / Moderate, Strong)  Methyldopa, labetalol and nifedipine are acceptable choices in the 1^st^ trimester of pregnancy  (II-2B / Low, Weak) |
| **Antihypertensives NOT to use during pregnancy** (and should be stopped) |  |  | For women with any HDP, ACE, ARBs or chlorothiazide (as they are associated with an increased risk of major malformations)  For women with chronic hypertension, stop ACE inhibitors or ARBs in pregnancy (preferably within 2 working days of notification of pregnancy) and offer alternatives  Tell women who took ACE inhibitors or ARBs “during pregnancy” that these medications increase the risk of congenital abnormalities  Tell women who took chlorothiazide “during pregnancy” that this medication may increase the risk of congenital abnormalities and neonatal complications |  | For women with any HDP, ACE inhibitors, ARBs, and direct renin inhibitors |  | For women with uncomplicated chronic hypertension, ACE inhibitors, ARBs, renin inhibitors, and mineralcorticoid receptor antagonists are NOT recommended  (Moderate, Strong) | For women with any HDP, atenolol and prazosin  (I-D / Moderate, Weak) – not acceptable for use  (I-D / Low, Weak) - stopping  For women with any HDP, ACE inhibitors and ARBs (which should be stopped)  (II-2E / Moderate, Strong) – not acceptable for use  (II-2D / Low, Weak) - stopping |
| **Antenatal corticosteroids** |  |  |  |  |  |  |  |  |
| **“≤ 34 weeks” – FIRST dose** |  |  | “Between 24 and 34 wks”  For women with PET who are likely to deliver within 7 days |  | “Before 34 weeks”  For women with any HDP who are likely to delivery within 2-10 days |  | “At ≤34^0^ wks”  For women with severe PET or superimposed PET who are receiving expectant care  (High, Strong) for severe PET  (High, Strong) for superimposed PET  “≤33 6/7 wks”  For women with severe PET who require delivery, without delivery being delayed  (Moderate, Strong)  NOTE: Listed were: uncontrollable severe hypertension, eclampsia, pulmonary edema, abruption placentae, disseminated intravascular coagulation, evidence of nonreassuring feta status, intrapartum fetal demise  “≤33 6/7 wks”  For women with severe PET who are stable enough to have delivery delayed by 48hr  (Moderate, Qualified)  NOTE: Criteria specified were: low platelet count (< 100,000/microliter), persistently abnormal hepatic enzyme concentrations (twice or more the upper normal values), fetal growth restriction (less than the fifth percentile), severe oligohydramnios (amniotic fluid index < 5cm), reversed end-diastolic flow on umbilical artery Dopppler studies, new-onset renal dysfunction or increasing renal dysfunction. | “At ≤34^6^ wks”  For women with PET  (I-A / High, Strong)  “≤346 wks”  For women with GH who may deliver within the next 7 days  (III-L / Low, Weak) |
| ***REPEAT*** *dosing* |  |  |  |  | “Before 33 wks”  For women with any HDP, ONLY if first does were given at <30 wks and > 14 days prior |  |  | “≤34^6^ wks”  For women with any HDP, if first dose ≥7 days prior  (I-C / Low, Weak) |
| **“35-36 wks”** |  |  | “35-36 wks”  For women with PET who are likely to deliver within 7 days |  |  |  |  |  |
| **≤ 38^6^ weeks gestation and elective Caesarean** |  |  |  |  |  |  |  | “≤ 38^6^ wks”  May consider for women with any HDP who are delivered by elective Caesarean  (I-B / Low, Weak) |
| **Timing of delivery** |  |  |  |  |  |  |  |  |
| **General comments** |  |  | For women with PET at “before 34 wks”, consultant obstetric staff should document maternal and fetal indications for elective birth |  | For women with any HDP, indications should be based on care provider’s own knowledge and experience |  |  | For women with “severe PET”, consultation must be undertaken (by telephone is necessary) with an obstetrician  (III-B / Low, Strong) |
| **Delivery indicated (indications)** |  |  | For women with any HDP (regardless of GA) who have refractory severe hypertension after BP has been controlled and a course of antenatal corticosteroids has been completed (if appropriate)  For women with PET “before 34 wks” who have a maternal or fetal indication for delivery (as specified by the care plan), after discussion with neonatal and anaesthetic teams, and after a course of antenatal corticosteroids has been “given”  For women with PET “after 37+0 wks” who have mild to moderate hypertension | For women with severe PET before fetal viability (and at a GA at which fetus not viable or unlikely to achieve viability in 1-2 wks)  (Very low, Strong)  For women with severe PET “before 34 wks” or “between 34 and 36 (+6 days) wks” who cannot be monitored or who have uncontrolled maternal hypertension, increasing maternal organ dysfunction or fetal distress  (Very low, Weak)  In women with mild GH or mild PET “at term” (Moderate, Weak)  For women with severe PET “at term”.  (Low, Strong) | For women with severe PET (including HELLP) or any HDP with an abnormal Doppler |  | For women with severe PET or HELLP syndrome before fetal viability (after maternal stabilization)  (Moderate, Strong) for severe PET  (High, Strong) for HELLP  For women with PET or superimposed PET at any GA who have unstable maternal or fetal conditions (after maternal stabilization)  (Moderate, Strong)  (Moderate, Strong)  NOTE: Listed were uncontrollable severe hypertension, eclampsia, pulmonary edema, abruption placentae, disseminated intravascular coagulation, nonreassuring fetal status  For women with severe PET or HELLP syndrome “≥34 0/7 wks”, or superimposed PET with severe features “beyond 34 0/7 wks” (after maternal stabilization)  (Moderate, Strong) severe PET  (Moderate, Strong) HELLP  (Moderate, Strong) superimposed PET  For women with mild GH or mild PET at “≥37 0/7 wks” who have no severe features  (Moderate, Qualified) | For women with uncomplicated chronic hypertension, consider delivery at 38 0/7 to 39 6/7 wks  (II-1B / Low, Weak)  For women with GH at ≥37 wks, delivery within days should be discussed  (I-B / Low, Weak)  For women with PET at <24^0^ wks, delivery should be discussed as an option  (II-2B / Low, Weak)  For women with “severe PET” regardless of GA  (III-C / Low, Strong)  For women with PET at ≥37 wks (I-A / High, Strong)  For women with HELLP at ≥35^0^ wks  (II-2B / Moderate, Strong) |
| **Expectant care ONLY until steroids have been administered** |  |  |  | For women with HELLP syndrome “from fetal viability to 33 6/7 wks” with stable maternal and fetal conditions  (Low, Qualified) |  |  |  | For women with HELLP syndrome at 24^0^-34^6^ wks  If there is temporary improvement in maternal laboratory testing  (II-2B / Low, Weak) |
| **Expectant care** |  |  | For women with PET “until 34 wks”  For women with chronic hypertension at <37 wks and BP <160/110mmHg  For women with GH “before 37 wks” who have BP <160/110 mmHg (even on antihypertensive treatment)  For women with PET at  34+0 to 36+6 weeks who have mild or moderate hypertension, depending on maternal and fetal condition, risk factors and availability of neonatal intensive care | For women with severe PET “before 34wk” who have a viable fetus and can be monitored  (Very low, Weak)  For women with severe PE “between 34 and 36 wks (+6 days)” who have a viable fetus and can be monitored  (Very low, Weak) |  |  | For women with severe PET or severe superimposed PET at <34 0/7 wks who have stable maternal and fetal conditions and who can be monitored at facilities with adequate intensive care resources  (Moderate, Strong) for PET  (Moderate, Strong) for superimposed PET  For women with superimposed PET “at <37 0/7 wks” who have no severe features and stable maternal and fetal conditions  (Low, Qualified)  For women with mild GH or PET at “<37 0/7 wks” who have no severe features or indication for delivery, and can be monitored  (Low, Qualified)  For women with uncomplicated chronic hypertension at <38 wks (Moderate, Strong)  For women with PET regardless of the amount or change in proteinuria  (Moderatre, Strong) | For women with non-severe PET at 24^0^-33^6^ wks, at centres capable of caring for very preterm infants  (I-B / Moderate, Weak) |
| ***Care plan*** |  |  | For women with severe GH or PET, write a care plan that includes: timing and mode of delivery, indications for delivery, timing of antenatal corticosteroids, and when discussion should take place with neonatology and obstetric anaesthesia |  |  |  |  |  |
| **Evidence insufficient to make a recommendation about delivery or expectant care** |  |  | For women with chronic hypertension at ≥37 wks and BP <160/110mmHg (“timing of birth and indications for birth to be agreed upon between woman and specialist”)  For women with GH “after 37 wks” who have BP <160/110mmHg (even on antihypertensive therapy) (“ timing of birth, and maternal and fetal indications for birth should be agreed between the woman and the senior obstetrician”) |  |  |  |  | For women with non-severe PET at 34^0^–36^6^ wks  (III-L / Low, Weak)  For women with GH at <37 wks  (III-L / Very low, Weak) |
| **Labour and delivery** |  |  |  |  |  |  |  |  |
| **Intrapartum care** |  |  | Advice and treatment should be in line with  ‘Intrapartum care: management and delivery of care to women in labour’ (NICE clinical guideline 55), unless it specifically differs from recommendations in this guideline |  |  |  |  |  |
| ***BP management*** |  |  | For women with any HDP, continue antihypertensive therapy  For women any HDP, monitor BP continuously in women who have severe hypertension, and hourly in women who have non-severe hypertension |  |  |  |  | For women with any HDP, continue antihypertensive therapy  (II-2B, / Low, Strong) |
| ***Investigations (for PET)*** |  |  | For women with any HDP and non-severe hypertension, perform haematological and biochemical tests using the same criteria as those used antenatally, whether or not regional anaesthesia is being considered |  |  |  |  | For women with PET, platelet count should be done upon admission to delivery suite  (II-1A / Low, Strong) |
| **Vaginal or Caesarean delivery** |  | For women with any HDP, Caesarean should be reserved for the usual obstetric indications  (II-2B)  If vaginal birth is planned and the cervix is unfavourable, cervical ripening is recommended  (I-A) | For women with any HDP and severe hypertenison, severe PET, or eclampsia, choice should be based on clinical circumstances and woman’s preference |  |  |  | For women with any HDP, Caesarean need not be the mode of delivery, depending on the GA, fetal presentation, cervical status and maternal and fetal conditions  (Moderate, Qualified) | For women with any HDP and evidence of fetal compromise, Caesarean delivery may be beneficial  (II-2B / Low, Strong)  For women with any HDP without fetal compromise, Caesarean should be reserved for the usual obstetric indications  (II-2B, / Low, Strong)  If vaginal birth is planned and the cervix is unfavourable, cervical ripening is recommended  (I-A / Moderate, Strong) |
| **Second stage (of labour)** |  |  | For women with any HDP with severe hypertension whose BP is not meeting treatment targets, recommend operative birth. Otherwise, do NOT limit second stage of labour |  |  |  |  |  |
| **Third stage** |  |  |  |  |  | For women with **any HDP**, active management with oxytocin recommended  (IA)  Ergonovine maleate should NOT be used to prevent/treat PPH if other suitable uterotonic drugs are available  (II-3D) |  | For women with any HDP, active management with oxytocin (5 units iv or 10units im) recommended  (I-A / Moderate, Strong)  Ergonovine maleate NOT be used to prevent/treat PPH  (II-3D / Low, Strong) |
| **Anaesthesia** |  |  |  |  |  |  |  |  |
| **General principles** |  |  |  |  |  |  | For women with PET, neuraxial analgesia or anaesthesia (spinal or epidural) is recommended  (Moderate, Strong) | For women with PET who are admitted to delivery suite, the anaesthesiologist should be informed  (II-3B / Low, Strong)  Early insertion of an epidural catheter for analgesia is recommended  (I-A / Moderate, Strong)  Acceptable methods of anaesthesia include epidural, spinal, combined spinal-epidural and general anaesthesia  (I-A / Moderate, Strong)  For women with any HDP, neuraxial analgesia and/or anaesthesia are appropriate:  a) With **PET**, provided there are no associated coagulation concerns.  (II-2E, / Low, Strong);  b) With a **platelet count ≥ 75 x 10^9^/L**  (II-2B / Very low, Weak);  c) Taking **low-dose ASA** in the presence of an adequate platelet count.  (I-A / Moderate/High, Strong);  d) Receiving UFH in a dose of ≤10,000 IU/d subcutaneously, 4 hr after the last dose and possibly IV after the last dose without any delay  (III-B / Very low, Weak);  e) Receiving UFH in a dose of 10,000 IU/d subcutaneously if they have a normal aPTT 4 hr after the last dose  (III-B / Very low, Weak);  f) Receiving **IV heparin** in a therapeutic dose if they have a normal aPTT 4 hr after the last dose  (III-B / Very low, Weak); or  g) Receiving **low-molecular weight heparin** (LMWH) a minimum of 10-12 hr after a prophylactic dose, or 24 hr after a therapeutic dose  (III-B / Very low, Weak)  For women with **any HDP**, phenylephrine or ephedrine may be used to treat hypotension during neuroaxial anaesthesia  (I-A / Moderate, Strong) |
| **Fluid administration** (including management of oliguria) |  |  | For women with severe PET, do NOT administer a fixed iv fluid bolus routinely prior to neuraxial analgesia  For women with severe PET, limit ongoing fluid administration to 80ml/hr (unless ongoing fluid losses) |  |  |  |  | For women with any HDP, do NOT administer a fixed iv fluid bolus routinely prior to neuraxial anaesthesia  (I-E / Low, Strong)  For women with PET, minimize iv and oral fluid intake  (II-2B / Low, Strong) |
| ***Treatment of oliguria*** |  |  |  |  |  |  |  | For women with any HDP, do NOT routinely administer fluid to treat oligura (<15mL/hr for 6 consecutive hours)  (III-D / Very low, Weak)  For women with any HDP, do NOT treat oliguria with dopamine or furosemide  (I-E / Moderate, Strong) |
| **Anesthesia – monitoring** |  |  |  |  |  |  |  |  |
| **Invasive haemodynamic monitoring** |  |  |  |  |  |  | For women with severe PET, do NOT routinely use invasive haemodynamic monitoring  (Low, Qualified) | For women with any HDP, do NOT routinely use central venous pressure monitoring  (II-2D / Very low/Low, Strong)  If a central venous monitoring is used, trends (and not absolute values) should be monitored  (II-2D / Very low/Low, Strong)  For women with any HDP, an arterial line may be used when BP is difficult to control or there is severe bleeding  (II-3B / Very low, Strong)  For women with any HDP, pulmonary artery catheterization is NOT recommended unless there is a specific indication  (II-D / Very low, Strong)  If used, a pulmonary catheter should be used only in a critical care setting  (III-B / Very low, Strong) |
| **Aspects of care for women with pre-existing hypertension** |  |  |  |  |  |  |  |  |
| **General considerations** |  |  | Advice and treatment should be in line with ‘Hypertension: the management of hypertension in adults in primary care’ (NICE clinical guideline 34), unless it specifically differs from recommendations in this guideline  Schedule additional antenatal consultations based on needs of woman and baby |  |  |  |  |  |
| **Specialist referral** |  |  | (Specialist in hypertensive disorders)  For women with secondary chronic hypertension |  |  |  |  |  |
| **Antihypertensive therapy – BEFORE pregnancy** |  | For women with any prior HDP, preconceptual advice should be offered at a formal postnatal review  (GPP) | Tell women of reproductive age who take ACE inhibitors or ARBs that these medications increase the risk of congenital abnormalities if they are taken “during pregnancy”  Tell women who take chlorothiazide that this medication may increase the risk of congenital abnormalities and neonatal complications if the drug is taken “during pregnancy”  Discuss alternatives to ACE inhibitors, ARBs, and chlorothiazide for women planning pregnancy |  | Discuss alternatives to ACE inhibitors, ARBs and direct renin inhibitors for women planning pregnancy |  | Women of reproductive age should not be prescribed ACE inhibitors, ARBs, renin inhibitors, and/or mineralocorticoid receptor antagonists unless there is a compelling indication  (Low, Qualified) | Pre-conceptual counselling is recommended  (III-C / Very low, Weak)  Discuss alternatives to ACE inhibitors and ARBs for women planning pregnancy  (II-2D / Low, Weak)  Changes to antihypertensive therapy should be made when planning pregnancy  (III-L / Very low, Weak) |
| **Aspects of care for women with preeclampsia** |  |  |  |  |  |  |  |  |
| **MgSO_4_** |  |  |  |  |  |  |  |  |
| **Indications** |  | Eclampsia (drug of first choice)  (I-A)  . | Eclampsia  Previous eclampsia in women with severe hypertension or severe PET in a critical care setting  Severe PET in a critical care setting when birth is planned within 24 hr  Severe PET  *NOTE: features listed: severe hypertension and proteinuria or mild or moderate hypertension and proteinuria with one or more of the following: symptoms of severe headache, problems with vision, such as blurring or flashing before the eyes, severe pain just below the ribs or vomiting, papilloedema, signs of clonus (≥ 3 beats), liver tenderness, HELLP syndrome, platelet count falling to below 100 × 109 per litre, abnormal liver enzymes (ALT or AST rising to above 70 IU/litre)* | Eclampsia (drug of first choice)  (Moderate/Strong)  Severe PET  (High, Strong) | Eclampsia (drug of first choice)  Severe PET  Mild/moderate PET (“can be considered”) |  | Eclampsia (drug of first choice)  (High, Strong)  Severe PET and superimposed PET with severe features, intrapartum and postpartum  (High, Strong) for severe PET  (Moderate, Strong) for superimposed PET with severe features  NOT routinely for PET with BP <160/110mmHg and no symptoms  (Low, Qualified)  Any PET introperatively during Caesarean delivery  (Moderate, Strong)  Postpartum, PET with severe hypertension or new-onset hypertension with headaches/blurred vision  (Low, Qualified) | Eclampsia (drug of first choice)  (I-A / High, Strong)  “Severe PET”  (I-A / High, Strong)  “Non-severe PET” (“can be considered based on cost considerations”)  (I-C / Moderate, Strong)  Fetal neuroprotection for women with any HDP when immiment preterm birth at  ≤31^6^ wks  (1-A / Moderate, Strong) |
| **Dosage** |  |  | Loading dose: 4g iv over 5 min  Maintenance dose: 1g/hour for 24 hr  Recurrent seizure dose: 2-4g iv over 5 min | “Full IV or IM” regimens (Moderate, Strong)  When full IV or IM regimens cannot be administered, administer loading dose and transfer immediately to a higher level health care facility (Very low, Weak) |  |  |  | Loading dose: “standard dosing”, usually 4g IV  (I-A / Moderate, Strong)  Maintenance dose: “standard dosing”, usually 1g/hr  (I-A / Moderate, Strong) |
| **Monitoring** |  |  |  |  | Monitor mothers according to local protocol |  |  | Do NOT routinely monitor serum Mg levels  (I-E / Low, Strong) |
| **Alternatives to MgSO4** |  |  | Do NOT use diazepam, phenytoin or lytic cocktail in preference to MgSO_4_ in women with eclampsia | Do NOT use diazepam, phenytoin or lytic cocktail in preference to MgSO_4_ in women with eclampsia or severe PET  (Moderate, Strong) for eclampsia  (High, Strong) severe PET |  |  |  | Do NOT use diazepam (I-E / Moderate, Strong) or phenytoin (I-E / High, Strong) in preference to MgSO_4_ in women with eclampsia or PET |
| **Plasma volume expansion** |  |  |  |  |  |  |  |  |
| **Preeclampsia** |  |  | NOT recommended for women with severe PET (unless hydralazine is the antenatal antihypertensive) |  |  |  |  | NOT recommended for women with PET  (I-E / Moderate, Strong) |
| **Therapies for HELLP** |  |  |  |  |  |  |  |  |
| **Platelet transfusion** |  |  |  |  |  |  |  | Platelet count < 20x10^9^/L  (III-B / Low, Strong)  Platelet count 20-49x10^9^/L prior to Caesarean  (III-B / Low, Strong)  Platelet count 20-49x10^9^/L prior to vaginal delivery if there is: excessive active bleeding, known platelet dysfunction, a rapidly falling platelet count, or coagulopathy  (II-2D / Low, Weak)  Platelet count ≥50x109/L if there is: excessive active bleeding, known platelet dysfunction, a rapidly falling platelet count, or coagulopathy.  (III-B, / Low, Weak)  Every obstetrical centre should be aware of the local delay between ordering and receiving platelets units  (III-B / Very low, Strong) |
| **Corticosteroids** |  |  | NOT recommended | NOT recommended  (Very low, Weak) |  |  | NOT recommended to improve clinical outcomes  (footnote)  Can be considered if improvement in platelet count would be useful (footnote) | NOT recommended  (II-3L / Moderate/Low, Weak) |
| **Plasma exchange or plasmapheresis** |  |  |  |  |  |  |  | NOT recommended  (II-3E, / Low, Strong) |
| **POSTPARTUM TREATMENT** |  |  |  |  |  |  |  |  |
| **Immediate postpartum management** |  |  |  |  |  |  |  |  |
| ***BP monitoring*** |  |  | For women with chronic hypertension or GH, measure BP daily for first 2 days, once/d on days 3-5, and as indicated if antihypertensive therapy is changed  For women with PET, measure BP 4x/day in hospital, once/d on days 3-5, and if abnormal then, on alternate days (until normal)  In women with PET who took antihypertensive therapy, measure BP 4x/day in hospital, then every 1-2 days for 2 wks until off treatment and normotensive |  |  | Inform women with any HDP that elevated BP may take time to resolve  (IIIA)  Inform women with GH that hypertension may worsen “during the postpartum period”  (IIIA) | For women with GH, PET, or superimposed PET, measure BP in hospital (or equivalent setting) for ≥72 hr and at some point on days 7-10 or earlier if PET symptoms occur  (Moderate, Qualified) | For women with any HDP, measure BP at some point on days 3-6 postpartum  (III-B / Low, Strong) |
| ***PET may appear or worsen*** |  | For women with PET, serial surveillance of maternal well-being is recommended  (II-3B) | For women with severe PET, ask about severe headache and epigastric pain when BP is measured  For women with PET with non-severe hypertension or those who have received critical care, measuring creatinine transaminases within 48-72 hr  If creatinine and transaminases are normal at 48-72hr after birth, they do NOT need to be retested  For women with PET, repeat platelet count, transaminases and serum creatinine “as clinically indicated” and at the 6-8 wk postnatal review  For women with PET who have stepped down from critical care (level 2),  do NOT measure fluid balance if creatinine is normal |  |  | Inform women with any HDP to report any symptoms or signs of PET  (IIIA) | Inform women with any HDP about symptoms and signs of PET which they should report immediately if they arise  (Low, Qualified) | Women with new/worsening postpartum hypertension should be evaluated for PET  (II-2B / Low, Weak)  For women with PET, there should be confirmation that end-organ dysfunction has resolved  (III-C / Very low, Strong) |
| **Antihypertensive therapy** |  |  |  |  |  |  |  |  |
| ***Continuation of antenatal antihypertensive therapy*** |  |  | For women with chronic hypertension,  continue antenatal antihypertensive therapy  In women with GH or PET who were taking antenatal antihypertensive therapy, continue therapy  If methyldopa was the antenatal antihypertensive, stop it within 2 days of birth. For women with chronic hypertension, restart the antihypertensive agent that was taken before planning pregnancy | For women with any HDP, continue antenatal antihypertensive therapy  (Very low, Strong) |  |  |  | For women with any HDP, especially with PET or preterm delivery, continue antihypertensive therapy  (II-2l / Low, Weak) |
| ***Treatment of severe hypertension*** |  |  | For women with any HDP, treat severe hypertension | For women with any HDP, treat severe hypertension with antihypertensive drugs  (Very low, Strong) |  |  | For women with any HDP, treat severe hypertension (BP ≥160/110mmHg) within one hour  (Low, Qualified) | For women with any HDP, treat severe hypertension with antihypertensive drugs  (I-A / Moderate, Strong)  For women with any HDP, goal of <160/110mmHg  (II-A / Moderate, Strong) |
| ***Treatment of non-severe hypertension*** |  |  | For women with “chronic hypertension”, goal of <140/90 mmHg  In women with GH or PET goal of <150/100mmHg  In women with GH or PET consider a reduced dose if BP <140/90mmHg. Reduce the dose if BP is <130/80mmHg |  |  |  | For women with any HDP goal of <150/100mmHg  (Low, Qualified) | For women with uncomplicated chronic hypertension, consider goal of <140/90mmHg  (III-l / Very low, Weak)  For women with chronic hypertension and co-morbidities other than pre-gestational diabetes mellitus, consider goal of <140/90mmHg  (III-C / Very low, Weak)  For women with chronic hypertension and pre-gestational diabetes mellitus, goal of <130/80mmHg  (III-C / Very low, Weak) |
| ***Antihypertensive agents and breastfeeding*** |  |  | Acceptable agents are nifedipine, labetalol, captopril, enalapril, atenolol and metoprolol  Do NOT prescribe diuretics to women who are breastfeeding or expressing milk  Insufficient evidence to comment on the neonatal safety of the following during breastfeeding: ACE inhibitors (other than enalapril and captopril), ARBs, and amlodipine |  |  |  |  | Acceptable agents are nifedipine XL, labetalol, captopril and enalapril, and  methyldopa (III-B / Moderate,Weak)  For women with any HDP postpartum, captopril, enalapril or quinapril may be used (III-B / Low, Weak) |
| **Discharge planning for community care** |  |  | For women with chronic hypertension,  review long-term antihypertensive treatment 2 weeks after the birth  Offer women with PET transfer to community care if they have no symptoms, BP <150/100mmHg, and laboratory abnormalities are stable/improving  For women with GH or PET, write a detailed care plan before transfer to community care  A care plan should include the following details: who will provide follow-up care, including medical review if needed, frequency of BP monitoring needed, thresholds for reducing or stopping treatment, indications for referral to primary care for BP review, and self-monitoring for symptoms |  |  | For women with any HDP, monitor BP at “all regular postpartum visits” in first 2 weeks postpartum, or until normal BP measured twice  (IIIB)  For women with any HDP who has an elevated BP upon discharge from hospital, ensure plan is in place for physician follow-up in the event that BP remains elevated (or increases further)  (III-B)  Upon discharge from midwifery care, communicate information about any HDP to the primary care provider  (IIIB) |  | For women with PET, there should be confirmation that end-organ dysfunction has resolved  (III-C / Very low, Strong) |
| **At midwifery visits between discharge and formal 6-8 wk postnatal review** |  |  | Offer medical review (with the pre-pregnancy team) at the 6-8 wks postnatal review for women with chronic hypertension  Offer medical review at the 6-8 wks postnatal review for women with GH or PET, especially if they are still on antihypertensive treatment 2 weeks after transfer to community care |  |  |  |  |  |
| **Formal medical postnatal review at 6-8 wks after delivery** |  |  | In women with PET, perform urinary reagent-strip testing. If proteinuria ≥1+, offer further review at 3 mos postpartum  If women with PET had improving but still abnormal haematological or biochemical indices at hospital discharge, repeat testing  For women with PET, do NOT routinely perform thrombophilia screening |  |  |  |  | For women with chronic hypertension or any HDP with persistent postpartum hypertension, perform the following (if not done previously): urinalysis, serum Na/K and creatinine, fasting glucose and lipid profile and standard ECG recommended  (III-l / Low, Weak)  For women with severe PET (particularly with presentation at <34 wks), screen for chronic hypertension and underlying renal disease)  (II-2B / Low, Weak)  For women with any HDP, consider screening for traditional cardiovascular risk markers  (II-2B / Low/Moderate, Weak) |
| ***Counselling about future pregnancy risks*** |  | For women with any HDP, offer preconceptual advice (GPP) |  |  |  |  |  | Advise women with any HDP to keep their BMI within healthy range to decrease risk in future pregnancy  (II-2A / Moderate, Strong) |
| ***Counselling about long-term health risks*** |  | For women with any HDP, offer “screening” and lifestyle counselling  (GPP) | Advise women with GH or PET (and their primary care physicians) that they are at increased risk of future hypertension and cardiovascular disease in later life  Advise women with PET with proteinuria (that has resolved) that they are still at increased risk kidney disease but the absolute risk is very low and follow-up is not necessary  Advise women with PET to keep their BMI within healthy range (18.5-24.8kg/m2, NICE clinical guideline 43) |  |  | Advise women with any HDP that they may be at increased risk of future hypertension and cardiovascular disease in later life  (III-B)  Advise women with any HDP of the benefits of a heart healthy diet and lifestyle  (IB) | For women with PET and preterm birth (<37 0/7 wks) or recurrent PET, consider yearly assessment of BP, lipids, fasting blood glucose and BMI  (Low, Qualified) | Advise women with any HDP to pursue a healthy diet and lifestyle  (I-B / Low, Weak) (6)  Advise women with any HDP to keep their BMI within healthy range for long-term health  (I-A / Low/Moderate, Strong) |
| **Specialist referral (e.g., renal, etc)** |  |  | Hypertension specialist - for women with GH or PET who still need antihypertensive therapy 6-8 wks after delivery  Kidney specialist - for women with PET who have proteinuria ≥1+ at 6-8 wks after delivery (although clinicians can reassess at 3mos post-delivery to confirm) |  |  |  |  | Offer in hospital specialist assessment with internal medicine - for women with any HDP when postpartum hypertension is difficult to control  (III-A / Low, Weak)  Offer outpatient renal assessment - for women who had PET who have proteinuiria, decreased eGFR (<60mL/min) or another indication of renal disease at 3-6 mos after delivery  (III-A / Low, Weak) |
| **NSAIDs** |  |  |  |  |  | For women with any HDP, limit use of NSAIDs and offer acetaminophen as an effective alternative (albeit with limited information about side effects)  (IIIL) |  | For women with any HDP, NSAIDs are NOT recommended if BP is difficult to control, there is kidney injury (oliguria and/or an elevated creatinine) (≥90 µM), or platelets are < 50x10^9^/L  (III-C / Low, Weak) |
| **Thromboprophylaxis** |  |  |  |  |  |  |  | Consider for women with PET, especially when there are other risk factors  (II-2B / Low, Weak) |

ACOG (American College of Obstetricians and Gynecologists), ACE inhibitor (angiotensin converting enzyme inhibitors), ALT (alanine aminotransaminase), AOM (Association of Ontario Midwives), ARB (angiotensin receptor blockers), ASA (aspirin), AST (aspartate aminotransferase), BP (blood pressure), Creat (creatinine), GA (gestational age), GH (gestational hypertension), BPP (good practice point), HDP (hypertensive disorders of pregnancy), HELLP (haemolysis, elevated liver enzyme, low platelet syndrome), LMWH (low molecular weight heparin), MgSO4 (magnesium sulphate), NICE (National Institute for Health and Clinical Excellence ), NVOG (Nederlandse Vereniging voor Obstetrie en Gynaecologie), PET (pre-eclampsia), PPH (postpartum haemorrhage), PRECOG (pre-eclampsia community guideline), QLD (Queensland Maternity and Neonatal Clinical Guidelines Program), SOGC (Society of Obstetricians and Gynaecologists of Canada), UFH (unfractionated heparin), WHO (World Health Organisation)

* Refer to Tables 2a and 2b for definitions of both the quality of the evidence and the strength of the recommendations as listed by individual guidelines. Yellow highlighting refers to information found in the footnotes of tables or in the text but linked with recommendations for easy identification.
