# Supplementary material for: Hypertensive Disorders of Pregnancy: A Systematic Review of International Clinical Practice Guidelines
Source: PLoS One. 2014 Dec 1;9(12):e113715. doi: 10.1371/journal.pone.0113715 (PMC4249974; doi:10.1371/journal.pone.0113715)
Supplement: Appendix S1 — Search strategy. (DOC) [file pone.0113715.s006.doc]

**Guidelines publication type *per se* without age limits**

Database(s): Ovid MEDLINE(R) In-Process & Other Non-Indexed Citations and Ovid MEDLINE(R) 1946 to Present
Search Strategy:

| **#** | **Searches** | **Results** |
| --- | --- | --- |
| 1 | exp Pregnancy/ | 713514 |
| 2 | exp Pregnancy Complications/ | 338434 |
| 3 | exp Pregnant Women/ | 5208 |
| 4 | exp Delivery, Obstetric/ | 60683 |
| 5 | exp Postpartum Period/ | 48132 |
| 6 | exp Peripartum Period/ | 274 |
| 7 | exp Gravidity/ | 717 |
| 8 | exp Maternal Welfare/ | 6119 |
| 9 | gravid*.mp. | 18417 |
| 10 | pregnan*.mp. | 784527 |
| 11 | gestation*.mp. | 180529 |
| 12 | cesarean*.mp. | 46084 |
| 13 | caesarean*.mp. | 14666 |
| 14 | obstetric*.mp. | 143377 |
| 15 | peripartum*.mp. | 2828 |
| 16 | intrapartum*.mp. | 6123 |
| 17 | postpartum*.mp. | 46971 |
| 18 | (child adj3 bearing).mp. | 2152 |
| 19 | childbearing.mp. | 9136 |
| 20 | matern*.mp. | 240070 |
| 21 | exp Hypertension/ | 210515 |
| 22 | exp Blood Pressure/ | 249479 |
| 23 | exp Blood Pressure Determination/ | 23888 |
| 24 | exp Antihypertensive Agents/ | 229610 |
| 25 | Hypertens*.mp. | 384119 |
| 26 | pre-eclamp*.mp. | 25758 |
| 27 | preeclamp*.mp. | 12702 |
| 28 | toxemia*.mp. | 6352 |
| 29 | toxaemia*.mp. | 1098 |
| 30 | gestosis.mp. | 1201 |
| 31 | antihypertensive*.mp. | 74273 |
| 32 | ((high$ or rais$ or elevat$ or heighten$ or increas$) adj3 (blood pressure or diastolic pressure or systolic pressure or pulse pressure)).mp. | 52141 |
| 33 | ((high$ or rais$ or elevat$ or heighten$ or increas$) adj3 (BP or DBP or SBP)).mp. | 13062 |
| 34 | or/21-33 | 745323 |
| 35 | exp Guidelines as Topic/ | 113271 |
| 36 | exp Guideline/ | 25073 |
| 37 | Guideline Adherence/ | 20381 |
| 38 | guideline*.mp. | 275651 |
| 39 | exp Consensus Development Conference/ | 9379 |
| 40 | practice parameter*.mp. | 895 |
| 41 | recommendation*.mp. | 146850 |
| 42 | consensus*.mp. | 115149 |
| 43 | standard*.mp. | 819863 |
| 44 | st.fs. | 547926 |
| 45 | protocol*.mp. | 376532 |
| 46 | position statement*.mp. | 2299 |
| 47 | position paper*.mp. | 2048 |
| 48 | good clinical practice.mp. | 1055 |
| 49 | implementation*.mp. | 123181 |
| 50 | (policy* or policies*).mp. | 219044 |
| 51 | reimbursement*.mp. | 31443 |
| 52 | performance measure*.mp. | 6698 |
| 53 | or/35-52 | 2188380 |
| 54 | (labor or laboring or labour or labouring).mp. | 113610 |
| 55 | or/1-20,54 | 1031970 |
| 56 | 34 and 53 and 55 | 4245 |
| 57 | (pregnan$ or gestation$ or matern$ or mother$ or pre-eclamp$ or preeclamp$ or pre eclamp$ or eclamp$ or HELLP or obstetric$ or postpartum$ or peripartum$ or intrapartum$ or antepartum$ or prepartum$ or antenatal$ or prenatal$ or postnatal$ or perinatal$ or internatal$ or cesarean$ or caesarean$ or childbearing or child-bearing or child bearing).ti. | 401316 |
| 58 | 56 and 57 | 2665 |
| 59 | limit 58 to yr="2003 -Current" | 1632 |
| 60 | diabet*.ti. | 235098 |
| 61 | 59 not 60 | 1532 |
| 62 | limit 61 to "review articles" | 381 |
| 63 | 61 not 62 | 1151 |
| 64 | limit 62 to (consensus development conference or consensus development conference, nih or guideline or practice guideline) | 20 |
| 65 | 63 or 64 | 1171 |
| 66 | limit 65 to (consensus development conference or consensus development conference, nih or guideline or practice guideline) | 47 |

Database(s): EBM Reviews - Cochrane Central Register of Controlled Trials August 2013
Search Strategy:

| **#** | **Searches** | **Results** |
| --- | --- | --- |
| 1 | exp Pregnancy/ | 14110 |
| 2 | exp Pregnancy Complications/ | 5725 |
| 3 | exp Pregnant Women/ | 58 |
| 4 | exp Delivery, Obstetric/ | 3410 |
| 5 | exp Postpartum Period/ | 894 |
| 6 | exp Peripartum Period/ | 4 |
| 7 | exp Gravidity/ | 31 |
| 8 | exp Maternal Welfare/ | 71 |
| 9 | gravid*.mp. | 367 |
| 10 | pregnan*.mp. | 19424 |
| 11 | gestation*.mp. | 6623 |
| 12 | cesarean*.mp. | 3702 |
| 13 | caesarean*.mp. | 1584 |
| 14 | obstetric*.mp. | 6007 |
| 15 | peripartum*.mp. | 79 |
| 16 | intrapartum*.mp. | 444 |
| 17 | postpartum*.mp. | 2482 |
| 18 | (child adj3 bearing).mp. | 66 |
| 19 | childbearing.mp. | 175 |
| 20 | matern*.mp. | 6642 |
| 21 | exp Hypertension/ | 12552 |
| 22 | exp Blood Pressure/ | 21658 |
| 23 | exp Blood Pressure Determination/ | 1655 |
| 24 | exp Antihypertensive Agents/ | 20762 |
| 25 | Hypertens*.mp. | 26328 |
| 26 | pre-eclamp*.mp. | 615 |
| 27 | preeclamp*.mp. | 514 |
| 28 | toxemia*.mp. | 59 |
| 29 | toxaemia*.mp. | 24 |
| 30 | gestosis.mp. | 24 |
| 31 | antihypertensive*.mp. | 10792 |
| 32 | ((high$ or rais$ or elevat$ or heighten$ or increas$) adj3 (blood pressure or diastolic pressure or systolic pressure or pulse pressure)).mp. | 5332 |
| 33 | ((high$ or rais$ or elevat$ or heighten$ or increas$) adj3 (BP or DBP or SBP)).mp. | 1600 |
| 34 | or/21-33 | 51371 |
| 35 | exp Guidelines as Topic/ | 1111 |
| 36 | exp Guideline/ | 0 |
| 37 | Guideline Adherence/ | 488 |
| 38 | guideline*.mp. | 5894 |
| 39 | exp Consensus Development Conference/ | 0 |
| 40 | practice parameter*.mp. | 10 |
| 41 | recommendation*.mp. | 3853 |
| 42 | consensus*.mp. | 1396 |
| 43 | standard*.mp. | 49475 |
| 44 | st.fs. | 8505 |
| 45 | protocol*.mp. | 32666 |
| 46 | position statement*.mp. | 10 |
| 47 | position paper*.mp. | 10 |
| 48 | good clinical practice.mp. | 91 |
| 49 | implementation*.mp. | 3362 |
| 50 | (policy* or policies*).mp. | 2392 |
| 51 | reimbursement*.mp. | 315 |
| 52 | performance measure*.mp. | 847 |
| 53 | or/35-52 | 94273 |
| 54 | (labor or laboring or labour or labouring).mp. | 6567 |
| 55 | or/1-20,54 | 29992 |
| 56 | 34 and 53 and 55 | 208 |
| 57 | (pregnan$ or gestation$ or matern$ or mother$ or pre-eclamp$ or preeclamp$ or pre eclamp$ or eclamp$ or HELLP or obstetric$ or postpartum$ or peripartum$ or intrapartum$ or antepartum$ or prepartum$ or antenatal$ or prenatal$ or postnatal$ or perinatal$ or internatal$ or cesarean$ or caesarean$ or childbearing or child-bearing or child bearing).ti. | 14833 |
| 58 | 56 and 57 | 130 |
| 59 | limit 58 to yr="2003 -Current" | 59 |
| 60 | diabet*.ti. | 16325 |
| 61 | 59 not 60 | 54 |
| 62 | limit 61 to "review articles" [Limit not valid; records were retained] | 54 |
| 63 | limit 61 to guideline | 0 |

Database(s): EBM Reviews - Cochrane Methodology Register 3rd Quarter 2012
Search Strategy:

| **#** | **Searches** | **Results** |
| --- | --- | --- |
| 1 | [exp Pregnancy/] | 0 |
| 2 | [exp Pregnancy Complications/] | 0 |
| 3 | [exp Pregnant Women/] | 0 |
| 4 | [exp Delivery, Obstetric/] | 0 |
| 5 | [exp Postpartum Period/] | 0 |
| 6 | [exp Peripartum Period/] | 0 |
| 7 | [exp Gravidity/] | 0 |
| 8 | [exp Maternal Welfare/] | 0 |
| 9 | gravid*.mp. | 1 |
| 10 | pregnan*.mp. | 166 |
| 11 | gestation*.mp. | 27 |
| 12 | cesarean*.mp. | 12 |
| 13 | caesarean*.mp. | 13 |
| 14 | obstetric*.mp. | 117 |
| 15 | peripartum*.mp. | 1 |
| 16 | intrapartum*.mp. | 9 |
| 17 | postpartum*.mp. | 14 |
| 18 | (child adj3 bearing).mp. | 1 |
| 19 | childbearing.mp. | 2 |
| 20 | matern*.mp. | 72 |
| 21 | [exp Hypertension/] | 0 |
| 22 | [exp Blood Pressure/] | 0 |
| 23 | [exp Blood Pressure Determination/] | 0 |
| 24 | [exp Antihypertensive Agents/] | 0 |
| 25 | Hypertens*.mp. | 147 |
| 26 | pre-eclamp*.mp. | 5 |
| 27 | preeclamp*.mp. | 8 |
| 28 | toxemia*.mp. | 0 |
| 29 | toxaemia*.mp. | 0 |
| 30 | gestosis.mp. | 0 |
| 31 | antihypertensive*.mp. | 47 |
| 32 | ((high$ or rais$ or elevat$ or heighten$ or increas$) adj3 (blood pressure or diastolic pressure or systolic pressure or pulse pressure)).mp. | 14 |
| 33 | ((high$ or rais$ or elevat$ or heighten$ or increas$) adj3 (BP or DBP or SBP)).mp. | 1 |
| 34 | or/21-33 | 188 |
| 35 | [exp Guidelines as Topic/] | 0 |
| 36 | [exp Guideline/] | 0 |
| 37 | Guideline Adherence/ | 0 |
| 38 | guideline*.mp. | 1071 |
| 39 | [exp Consensus Development Conference/] | 0 |
| 40 | practice parameter*.mp. | 1 |
| 41 | recommendation*.mp. | 807 |
| 42 | consensus*.mp. | 420 |
| 43 | standard*.mp. | 2132 |
| 44 | st.fs. | 0 |
| 45 | protocol*.mp. | 783 |
| 46 | position statement*.mp. | 0 |
| 47 | position paper*.mp. | 2 |
| 48 | good clinical practice.mp. | 15 |
| 49 | implementation*.mp. | 392 |
| 50 | (policy* or policies*).mp. | 757 |
| 51 | reimbursement*.mp. | 38 |
| 52 | performance measure*.mp. | 13 |
| 53 | or/35-52 | 4741 |
| 54 | (labor or laboring or labour or labouring).mp. | 65 |
| 55 | or/1-20,54 | 370 |
| 56 | 34 and 53 and 55 | 9 |
| 57 | (pregnan$ or gestation$ or matern$ or mother$ or pre-eclamp$ or preeclamp$ or pre eclamp$ or eclamp$ or HELLP or obstetric$ or postpartum$ or peripartum$ or intrapartum$ or antepartum$ or prepartum$ or antenatal$ or prenatal$ or postnatal$ or perinatal$ or internatal$ or cesarean$ or caesarean$ or childbearing or child-bearing or child bearing).ti. | 188 |
| 58 | 56 and 57 | 3 |
| 59 | limit 58 to yr="2003 -Current" | 2 |
| 60 | diabet*.ti. | 69 |
| 61 | 59 not 60 | 2 |
| 62 | limit 61 to "review articles" | 0 |
| 63 | limit 61 to guideline [Limit not valid; records were retained] | 2 |

**Database(s): EBM Reviews - Database of Abstracts of Reviews of Effects 3rd Quarter 2013**Search Strategy:

| **#** | **Searches** | **Results** |
| --- | --- | --- |
| 1 | [exp Pregnancy/] | 0 |
| 2 | [exp Pregnancy Complications/] | 0 |
| 3 | [exp Pregnant Women/] | 0 |
| 4 | [exp Delivery, Obstetric/] | 0 |
| 5 | [exp Postpartum Period/] | 0 |
| 6 | [exp Peripartum Period/] | 0 |
| 7 | [exp Gravidity/] | 0 |
| 8 | [exp Maternal Welfare/] | 0 |
| 9 | gravid*.mp. | 7 |
| 10 | pregnan*.mp. | 1461 |
| 11 | gestation*.mp. | 393 |
| 12 | cesarean*.mp. | 117 |
| 13 | caesarean*.mp. | 200 |
| 14 | obstetric*.mp. | 356 |
| 15 | peripartum*.mp. | 10 |
| 16 | intrapartum*.mp. | 31 |
| 17 | postpartum*.mp. | 176 |
| 18 | (child adj3 bearing).mp. | 4 |
| 19 | childbearing.mp. | 10 |
| 20 | matern*.mp. | 378 |
| 21 | [exp Hypertension/] | 0 |
| 22 | [exp Blood Pressure/] | 0 |
| 23 | [exp Blood Pressure Determination/] | 0 |
| 24 | [exp Antihypertensive Agents/] | 0 |
| 25 | Hypertens*.mp. | 998 |
| 26 | pre-eclamp*.mp. | 88 |
| 27 | preeclamp*.mp. | 30 |
| 28 | toxemia*.mp. | 0 |
| 29 | toxaemia*.mp. | 2 |
| 30 | gestosis.mp. | 0 |
| 31 | antihypertensive*.mp. | 308 |
| 32 | ((high$ or rais$ or elevat$ or heighten$ or increas$) adj3 (blood pressure or diastolic pressure or systolic pressure or pulse pressure)).mp. | 119 |
| 33 | ((high$ or rais$ or elevat$ or heighten$ or increas$) adj3 (BP or DBP or SBP)).mp. | 33 |
| 34 | or/21-33 | 1137 |
| 35 | [exp Guidelines as Topic/] | 0 |
| 36 | [exp Guideline/] | 0 |
| 37 | [Guideline Adherence/] | 0 |
| 38 | guideline*.mp. | 1503 |
| 39 | [exp Consensus Development Conference/] | 0 |
| 40 | practice parameter*.mp. | 19 |
| 41 | recommendation*.mp. | 1886 |
| 42 | consensus*.mp. | 3786 |
| 43 | standard*.mp. | 8007 |
| 44 | [st.fs.] | 0 |
| 45 | protocol*.mp. | 2607 |
| 46 | position statement*.mp. | 7 |
| 47 | position paper*.mp. | 10 |
| 48 | good clinical practice.mp. | 6 |
| 49 | implementation*.mp. | 422 |
| 50 | (policy* or policies*).mp. | 436 |
| 51 | reimbursement*.mp. | 31 |
| 52 | performance measure*.mp. | 36 |
| 53 | or/35-52 | 11334 |
| 54 | (labor or laboring or labour or labouring).mp. | 292 |
| 55 | or/1-20,54 | 1794 |
| 56 | 34 and 53 and 55 | 103 |
| 57 | (pregnan$ or gestation$ or matern$ or mother$ or pre-eclamp$ or preeclamp$ or pre eclamp$ or eclamp$ or HELLP or obstetric$ or postpartum$ or peripartum$ or intrapartum$ or antepartum$ or prepartum$ or antenatal$ or prenatal$ or postnatal$ or perinatal$ or internatal$ or cesarean$ or caesarean$ or childbearing or child-bearing or child bearing).ti. | 697 |
| 58 | 56 and 57 | 61 |
| 59 | limit 58 to yr="2003 -Current" [Limit not valid; records were retained] | 61 |
| 60 | diabet*.ti. | 671 |
| 61 | 59 not 60 | 56 |
| 62 | limit 61 to "review articles" [Limit not valid; records were retained] | 56 |
| 63 | limit 61 to guideline [Limit not valid; records were retained] | 56 |

**Same as above for EBM Reviews- Health Technology Assessments**

**Database(s): Embase 1974 to 2013 September 17**Search Strategy:

| 151 | exp pregnancy/ | 618869 |
| --- | --- | --- |
| 152 | exp pregnancy complication/ | 111295 |
| 153 | exp delivery/ | 118813 |
| 154 | exp puerperium/ | 40950 |
| 155 | exp perinatal period/ | 20471 |
| 156 | exp maternal welfare/ | 9984 |
| 157 | gravid*.mp. | 21855 |
| 158 | pregnan*.mp. | 780211 |
| 159 | gestation*.mp. | 211670 |
| 160 | cesarean*.mp. | 68005 |
| 161 | caesarean*.mp. | 20551 |
| 162 | obstetric*.mp. | 122887 |
| 163 | peripartum*.mp. | 3558 |
| 164 | intrapartum*.mp. | 7882 |
| 165 | postpartum*.mp. | 43194 |
| 166 | (child adj3 bearing).mp. | 2754 |
| 167 | childbearing.mp. | 9586 |
| 168 | matern*.mp. | 264279 |
| 169 | (labor or labour or laboring or labouring).mp. | 133431 |
| 170 | 151 or 152 or 153 or 154 or 155 or 156 or 157 or 158 or 159 or 160 or 161 or 162 or 163 or 164 or 165 or 166 or 167 or 168 or 169 | 1110006 |
| 171 | exp hypertension/ | 471853 |
| 172 | exp blood pressure/ | 386808 |
| 173 | exp blood pressure measurement/ | 57098 |
| 174 | exp antihypertensive agent/ | 566043 |
| 175 | Hypertens*.mp. | 615008 |
| 176 | pre-eclamp*.mp. | 10316 |
| 177 | preeclamp*.mp. | 38298 |
| 178 | toxemia*.mp. | 8617 |
| 179 | toxaemia*.mp. | 1002 |
| 180 | gestosis.mp. | 1579 |
| 181 | antihypertensive*.mp. | 101832 |
| 182 | [or/21-33] | 0 |
| 183 | [or/35-44] | 0 |
| 184 | [limit 50 to "review"] | 0 |
| 185 | [limit 56 to (human and yr="2003 -Current")] | 0 |
| 186 | [limit 57 to (dutch or english or french or german)] | 0 |
| 187 | exp pregnancy/ | 618869 |
| 188 | exp pregnancy complication/ | 111295 |
| 189 | exp delivery/ | 118813 |
| 190 | exp puerperium/ | 40950 |
| 191 | exp perinatal period/ | 20471 |
| 192 | exp maternal welfare/ | 9984 |
| 193 | gravid*.mp. | 21855 |
| 194 | pregnan*.mp. | 780211 |
| 195 | gestation*.mp. | 211670 |
| 196 | cesarean*.mp. | 68005 |
| 197 | caesarean*.mp. | 20551 |
| 198 | obstetric*.mp. | 122887 |
| 199 | peripartum*.mp. | 3558 |
| 200 | intrapartum*.mp. | 7882 |
| 201 | postpartum*.mp. | 43194 |
| 202 | (child adj3 bearing).mp. | 2754 |
| 203 | childbearing.mp. | 9586 |
| 204 | matern*.mp. | 264279 |
| 205 | (labor or labour or laboring or labouring).mp. | 133431 |
| 206 | 187 or 188 or 189 or 190 or 191 or 192 or 193 or 194 or 195 or 196 or 197 or 198 or 199 or 200 or 201 or 202 or 203 or 204 or 205 | 1110006 |
| 207 | exp hypertension/ | 471853 |
| 208 | exp blood pressure/ | 386808 |
| 209 | exp blood pressure measurement/ | 57098 |
| 210 | exp antihypertensive agent/ | 566043 |
| 211 | Hypertens*.mp. | 615008 |
| 212 | pre-eclamp*.mp. | 10316 |
| 213 | preeclamp*.mp. | 38298 |
| 214 | toxemia*.mp. | 8617 |
| 215 | toxaemia*.mp. | 1002 |
| 216 | gestosis.mp. | 1579 |
| 217 | antihypertensive*.mp. | 101832 |
| 218 | ((high$ or rais$ or elevat$ or heighten$ or increas$) adj3 (blood pressure or diastolic pressure or systolic pressure or pulse pressure)).mp. | 72313 |
| 219 | ((high$ or rais$ or elevat$ or heighten$ or increas$) adj3 (BP or DBP or SBP)).mp. | 17389 |
| 220 | or/207-219 | 1321465 |
| 221 | exp practice guideline/ | 304651 |
| 222 | guideline*.mp. | 389283 |
| 223 | practice parameter*.mp. | 1139 |
| 224 | recommendation*.mp. | 187202 |
| 225 | consensus*.mp. | 131560 |
| 226 | standard*.mp. | 1333710 |
| 227 | protocol*.mp. | 369334 |
| 228 | position statement*.mp. | 2670 |
| 229 | position paper*.mp. | 2410 |
| 230 | good clinical practice.mp. | 7758 |
| 231 | or/221-230 | 2139269 |
| 232 | 206 and 220 and 231 | 7566 |
| 233 | (pregnan$ or gestation$ or matern$ or mother$ or pre-eclamp$ or preeclamp$ or pre eclamp$ or eclamp$ or HELLP or obstetric$ or postpartum$ or peripartum$ or intrapartum$ or antepartum$ or prepartum$ or antenatal$ or prenatal$ or postnatal$ or perinatal$ or internatal$ or cesarean$ or caesarean$ or childbearing or child-bearing or child bearing).ti. | 473273 |
| 234 | 232 and 233 | 4215 |
| 235 | diabet*.ti. | 301493 |
| 236 | 234 not 235 | 3955 |
| 237 | limit 236 to "review" | 761 |
| 238 | 236 not 237 | 3194 |
| 239 | exp practice guideline/ | 304651 |
| 240 | 236 and 239 | 718 |
| 241 | 237 and 239 | 260 |
| 242 | 240 or 241 | 718 |
| 243 | limit 242 to (human and yr="2003 -Current") | 538 |
| 244 | limit 243 to (dutch or english or french or german) | 498 |
| 245 | practice guideline/ or exp clinical pathway/ or exp clinical protocol/ or exp consensus development/ or exp good clinical practice/ | 303593 |
| 246 | 238 and 245 | 458 |
| 247 | 237 and 245 | 260 |
| 248 | 246 or 247 | 718 |
| 249 | limit 248 to (human and yr="2003 -Current") | 538 |
| 250 | limit 249 to (dutch or english or french or german) | 498 |
| 251 | limit 250 to exclude medline journals | 66 |
